# Posicionamento sobre Emergências Cardiovasculares para Eventos Esportivos – 2026

**DOI:** 10.36660/abc.20260220

**Published:** 2026-04-28

**Authors:** Luiz Eduardo Fonteles Ritt, Antonio Carlos Avanza, Ricardo Stein, Rodrigo Otavio Bougleux Alô, Marcelo Bichels Leitão, Arthur Haddad Herdy, Marconi Gomes, Carolina Christianini Mizzaci, Anderson Donelli da Silveira, Fabio de Freitas Guimarães Guerra, Fabricio Braga, Milena dos Santos Barros Campos, Ricardo Galesso Cardoso, Nivaldo Filgueiras, Mateus Freitas Teixeira, Marcos Machado Barojas, Cléa Simone Sabino de Souza Colombo, Luciana Diniz Nagem Janot de Matos

**Affiliations:** 1 Escola Bahiana de Medicina e Saúde Pública Salvador BA Brasil Escola Bahiana de Medicina e Saúde Pública, Salvador, BA – Brasil; 2 Instituto D’Or de Pesquisa e Ensino Salvador BA Brasil Instituto D’Or de Pesquisa e Ensino, Salvador, BA – Brasil; 3 Hospital Cárdio Pulmonar Salvador BA Brasil Hospital Cárdio Pulmonar, Salvador, BA – Brasil; 4 Universidade Vila Velha ES Brasil Universidade Vila Velha, ES – Brasil; 5 Universidade Federal do Rio Grande do Sul RS Brasil Universidade Federal do Rio Grande do Sul, RS – Brasil; 6 Instituto Dante Pazzanese de Cardiologia São Paulo SP Brasil Instituto Dante Pazzanese de Cardiologia, São Paulo - SP – Brasil; 7 IEMEX - Instituto de Endocrinologia e Medicina do Exercício Curitiba PR Brasil IEMEX - Instituto de Endocrinologia e Medicina do Exercício, Curitiba, PR – Brasil; 8 Instituto de Cardiologia de Santa Catarina Florianópolis SC Brasil Instituto de Cardiologia de Santa Catarina, Florianópolis, SC – Brasil; 9 Prefeitura de Belo Horizonte Belo Horizonte MG Brasil Prefeitura de Belo Horizonte, Belo Horizonte, MG – Brasil; 10 Hospital de Clinicas de Porto Alegre Porto Alegre RS Brasil Hospital de Clinicas de Porto Alegre, Porto Alegre, RS – Brasil; 11 Programa de Pós-Graduação em Cardiologia Universidade Federal do Rio Grande do Sul Porto Alegre RS Brasil Programa de Pós-Graduação em Cardiologia Universidade Federal do Rio Grande do Sul, Porto Alegre, RS – Brasil; 12 Hospital Moinhos de Vento Porto Alegre RS Brasil Hospital Moinhos de Vento, Porto Alegre, RS – Brasil; 13 Hospital Santa Casa de Santos Santos SP Brasil Hospital Santa Casa de Santos, Santos, SP – Brasil; 14 Universidade Metropolitana de Santos Santos SP Brasil Universidade Metropolitana de Santos, Santos, SP – Brasil; 15 Laboratório de Performance Humana Rio de Janeiro RJ Brasil Laboratório de Performance Humana, Rio de Janeiro, RJ – Brasil; 16 "Hospital Universitário de Sergipe Aracajú SE Brasil "Hospital Universitário de Sergipe, Aracajú, SE – Brasil; 17 Hospital São Lucas Rede D’Or São Luiz Aracajú SE Brasil Hospital São Lucas Rede D’Or São Luiz, Aracajú, SE – Brasil; 18 Einstein Hospital Israelita São Paulo SP Brasil Einstein Hospital Israelita, São Paulo, SP – Brasil; 19 Universidade do Estado da Bahia Salvador BA Brasil Universidade do Estado da Bahia, Salvador, BA – Brasil; 20 Clínica Fit Center e Club de Regatas Vasco da Gama Niterói RJ Brasil Clínica Fit Center e Club de Regatas Vasco da Gama, Niterói, RJ – Brasil; 21 Hospital Português Salvador BA Brasil Hospital Português, Salvador, BA – Brasil; 22 Faculdade de Medicina São Leopoldo Mandic Campinas SP Brasil Faculdade de Medicina São Leopoldo Mandic, Campinas, SP – Brasil

**Table:** 

Posicionamento sobre Emergências Cardiovasculares para Eventos Esportivos – 2026	
O relatório abaixo lista as declarações de interesse conforme relatadas à SBC pelos especialistas durante o período de desenvolvimento deste posicionamento, 2025/2026.	
Especialista	Tipo de relacionamento com a indústria
Anderson Donelli da Silveira	Nada a ser declarado
Antonio Carlos Avanza Junior	Nada a ser declarado
Arthur Haddad Herdy	Declaração financeira B - Financiamento de pesquisas sob sua responsabilidade direta/pessoal (direcionado ao departamento ou instituição) provenientes da indústria farmacêutica, de órteses, próteses, equipamentos e implantes, brasileiras ou estrangeiras: Jansen: Melvexian; Novartis: Inclisiran.
Carolina Christianini Mizzaci	Nada a ser declarado
Cléa Simone Sabino de Souza Colombo	Nada a ser declarado
Fabio de Freitas Guimarães Guerra	Declaração financeira A - Pagamento de qualquer espécie e desde que economicamente apreciáveis, feitos a (i) você, (ii) ao seu cônjuge/ companheiro ou a qualquer outro membro que resida com você, (iii) a qualquer pessoa jurídica em que qualquer destes seja controlador, sócio, acionista ou participante, de forma direta ou indireta, recebimento por palestras, aulas, atuação como proctor de treinamentos, remunerações, honorários pagos por participações em conselhos consultivos, de investigadores, ou outros comitês, etc. Provenientes da indústria farmacêutica, de órteses, próteses, equipamentos e implantes, brasileiras ou estrangeiras: - Astrazeneca: Forxiga, Lokelma, Breztri. Outros relacionamentos Financiamento de atividades de educação médica continuada, incluindo viagens, hospedagens e inscrições para congressos e cursos, provenientes da indústria farmacêutica, de órteses, próteses, equipamentos e implantes, brasileiras ou estrangeiras: - Novonordisk: Rybelsus.
Fabricio Braga	Nada a ser declarado
Luciana Janot	Nada a ser declarado
Luiz Eduardo Fonteles Ritt	Declaração financeira A - Pagamento de qualquer espécie e desde que economicamente apreciáveis, feitos a (i) você, (ii) ao seu cônjuge/ companheiro ou a qualquer outro membro que resida com você, (iii) a qualquer pessoa jurídica em que qualquer destes seja controlador, sócio, acionista ou participante, de forma direta ou indireta, recebimento por palestras, aulas, atuação como proctor de treinamentos, remunerações, honorários pagos por participações em conselhos consultivos, de investigadores, ou outros comitês, etc. Provenientes da indústria farmacêutica, de órteses, próteses, equipamentos e implantes, brasileiras ou estrangeiras: - Pfizer: amiloidose; Astrazenca: amiloidose; Pesquisa Clínica em lípides, Insuficiência cardíaca, dislipidemia: Astrazeneca, Novartis, Novo Nordisk, Boeringher. Outros financimaentos - Financiamento de atividades de educação médica continuada, incluindo viagens, hospedagens e inscrições para congressos e cursos, provenientes da indústria farmacêutica, de órteses, próteses, equipamentos e implantes, brasileiras ou estrangeiras. Astrazeneca: amiloidose, Pfizer: amiloidose
Marcelo Bichels Leitão	Nada a ser declarado
Marconi Gomes	Nada a ser declarado
Marcos Machado Barojas	Nada a ser declarado
Mateus Freitas Teixeira	Nada a ser declarado
Milena dos Santos Barros Campos	Nada a ser declarado
Nivaldo Filgueiras	Declaração financeira A - Pagamento de qualquer espécie e desde que economicamente apreciáveis, feitos a (i) você, (ii) ao seu cônjuge/ companheiro ou a qualquer outro membro que resida com você, (iii) a qualquer pessoa jurídica em que qualquer destes seja controlador, sócio, acionista ou participante, de forma direta ou indireta, recebimento por palestras, aulas, atuação como proctor de treinamentos, remunerações, honorários pagos por participações em conselhos consultivos, de investigadores, ou outros comitês, etc. Provenientes da indústria farmacêutica, de órteses, próteses, equipamentos e implantes, brasileiras ou estrangeiras: - AstraZeneca: Lokelma, Forxiga; Servier: Adesão/Acertanlo, Acertil; Boehringer: Jardiance. Outros relacionamentos Financiamento de atividades de educação médica continuada, incluindo viagens, hospedagens e inscrições para congressos e cursos, provenientes da indústria farmacêutica, de órteses, próteses, equipamentos e implantes, brasileiras ou estrangeiras: - Servier: Acertanlo; AstraZeneca: Lokelma.
Ricardo Galesso Cardoso	Nada a ser declarado
Ricardo Stein	Declaração financeira A - Pagamento de qualquer espécie e desde que economicamente apreciáveis, feitos a (i) você, (ii) ao seu cônjuge/ companheiro ou a qualquer outro membro que resida com você, (iii) a qualquer pessoa jurídica em que qualquer destes seja controlador, sócio, acionista ou participante, de forma direta ou indireta, recebimento por palestras, aulas, atuação como proctor de treinamentos, remunerações, honorários pagos por participações em conselhos consultivos, de investigadores, ou outros comitês, etc. Provenientes da indústria farmacêutica, de órteses, próteses, equipamentos e implantes, brasileiras ou estrangeiras: - Life Genomics: TEB. B - Financiamento de pesquisas sob sua responsabilidade direta/pessoal (direcionado ao departamento ou instituição) provenientes da indústria farmacêutica, de órteses, próteses, equipamentos e implantes, brasileiras ou estrangeiras: - CNPq: FIPE/HCPA.
Rodrigo Otavio Bougleux Alô	Declaração financeira A - Pagamento de qualquer espécie e desde que economicamente apreciáveis, feitos a (i) você, (ii) ao seu cônjuge/ companheiro ou a qualquer outro membro que resida com você, (iii) a qualquer pessoa jurídica em que qualquer destes seja controlador, sócio, acionista ou participante, de forma direta ou indireta, recebimento por palestras, aulas, atuação como proctor de treinamentos, remunerações, honorários pagos por participações em conselhos consultivos, de investigadores, ou outros comitês, etc. Provenientes da indústria farmacêutica, de órteses, próteses, equipamentos e implantes, brasileiras ou estrangeiras: - Libbs: Pitavastatina. Outros relacionamentos Financiamento de atividades de educação médica continuada, incluindo viagens, hospedagens e inscrições para congressos e cursos, provenientes da indústria farmacêutica, de órteses, próteses, equipamentos e implantes, brasileiras ou estrangeiras. - Sanofi: congresso.

## Sumário

**1. Introdução: Morte Súbita no Esporte – Escopo do Problema** 5
  **1.1. Etiologias** 5
  **1.2. Epidemiologia e Incidência** 7
  **1.3. Situação Atual: Lacunas entre Conhecimento e Prática** 7
  **1.4. Justificativa e Chamada à Ação** 7
**2. Avaliação Pré-Participação em Cardiologia do Esporte** 8
  **2.1. Contexto Brasileiro** 8
  **2.2. Recomendações da Sociedade Brasileira de Cardiologia para a Avaliação Pré-Participação** 8
  **2.3. Perfil e Padrão de Avaliação Recomendada** 8
**3. Checklist e Equipamentos Essenciais para o Atendimento de Emergências Cardiovasculares em Grandes Eventos Esportivos** 8
  **3.1. Estrutura do Plano de Equipamentos** 9
  **3.2. Equipamentos de Suporte Básico de Vida** 9
  **3.3. Equipamentos de Suporte Avançado de Vida** 10
  **3.4. Comunicação e Logística Operacional** 10
  **3.5. Equipamentos Específicos por Modalidade Esportiva** 10
  **3.6. Procedimentos de Inspeção, Manutenção e Treinamento** 11
  **3.7. Considerações Finais** 11
**4. Componentes da Equipe, Formação e Capacitação** 11
**5. Equipamentos, Localização e Logística em Eventos Esportivos** 13
  **5.1. Introdução** 13
  **5.2. Equipamentos Essenciais** 13
    **5.2.1. Desfibriladores Externos Automáticos** 13
    **5.2.2. Material de Suporte Avançado** 13
    **5.2.3. Recursos Complementares** 13
  **5.3. Localização e Infraestrutura de Atendimento** 13
    **5.3.1. Mapeamento Interno** 13
    **5.3.2. Salas Médicas e Postos de Atendimento** 13
    **5.3.3. Transporte Interno e Externo** 13
  **5.4. Logística Operacional** 14
    **5.4.1. Plano de Ação de Emergência** 14
    **5.4.2. Equipe e Treinamento** 14
    **5.4.3. Comunicação e Central de Comando** 14
  **5.5. Impacto dos Equipamentos e da Logística sobre a Sobrevida** 14
  **5.6. Considerações Operacionais para o Contexto Brasileiro** 14
  **5.7. Conclusão** 14
**6. Logística de Atendimento, Evacuação e Transporte** 15
  **6.1. Planejamento e Infraestrutura Médica** 15
    **6.1.1. Avaliação de Risco Pré-Evento** 15
    **6.1.2. Estrutura Assistencial** 16
    **6.1.3. Comunicação e Comando** 16
    **6.1.4. Operação no Evento** 16
    **6.1.5. Postos Médicos Avançados** 16
    **6.1.6. Equipes Móveis** 17
    **6.1.7. Comunicação e Centro de Comando** 17
  **6.2. Atendimento Médico e Triagem** 18
    **6.2.1. Logística Durante o Atendimento Médico** 18
    **6.2.2. Registro e Indicadores de Qualidade** 18
  **6.3. Evacuação Médica** 18
    **6.3.1. Critérios de Evacuação** 18
    **6.3.2. Rotas de Evacuação** 18
    **6.3.3. Integração com Hospitais de Referência** 18
    **6.3.4. Transporte Seguro** 18
    **6.3.5. Monitorização Durante o Transporte** 18
  **6.4. Aspectos Legais e de Segurança** 19
  **6.5. Considerações por Ambiente** 19
**7. Protocolos de Atendimento Básico e Avançado (a Cadeia de Sobrevivência) Adaptados ao Esporte** 19
  **7.1. Reconhecimento Imediato e Ativação do Serviço de Emergência** 19
  **7.2. Reanimação Cardiopulmonar Precoce de Alta Qualidade** 19
  **7.3. Desfibrilação Rápida** 19
  **7.4. Suporte Avançado de Vida e Cuidados Pós-Parada** 19
  **7.5. Protocolo Específico para o Local de Treino/Competição** 20
**Referências** 21

## 1. Introdução: Morte Súbita no Esporte – Escopo do Problema

A morte súbita é tradicionalmente caracterizada como um episódio fatal inesperado, independentemente de ter sido presenciado, que ocorre até 1 hora após o início dos sintomas ou dentro das primeiras 24 horas após o indivíduo ter sido visto previamente em boas condições e sem queixas clínicas.^1^ Quando a morte súbita acontece no contexto esportivo, considera-se sua relação com a atividade física quando o evento ocorre até 1 hora após a realização de exercícios de intensidade moderada ou elevada.^2^ Em atletas, frequentemente vistos como ícones de saúde, sua ocorrência assume repercussões emocionais e sociais marcantes, afetando familiares, equipes, espectadores e instituições esportivas. A complexidade do tema decorre das múltiplas variáveis envolvidas, incluindo diferenças de sexo, idade, etnia, características corporais, modalidade praticada, posição esportiva, intensidade dos treinamentos e fatores ambientais.

### 1.1. Etiologias

As causas de morte súbita em atletas podem ser congênitas ou adquiridas, predominando, nos indivíduos mais jovens (< 35 anos), as cardiopatias estruturais genéticas e as canalopatias, além das miocardites de origem viral.^3-6^ Em atletas com 35 anos ou mais, a doença arterial coronariana (DAC) corresponde à etiologia responsável por mais de 80% dos casos de morte súbita cardíaca (MSC).^7-9^

Em algumas situações, a autópsia revela um coração estruturalmente normal, caracterizando a chamada síndrome da morte súbita arrítmica (SADS, do inglês, *sudden arrhythmic death syndrome*).^3-5^ Dados recentes de registros dos EUA, Reino Unido e Austrália sugerem que a SADS tem sido relatada como causa frequente de morte súbita em atletas jovens.^6^ Ainda assim, doenças estruturais como a miocardiopatia hipertrófica (MCH) e a cardiomiopatia arritmogênica ventricular (CAV) permanecem entre as principais etiologias associadas à morte súbita nessa população. Entre as causas adquiridas, destaca-se a miocardite viral, condição crescente entre atletas jovens, frequentemente relacionada a vírus comuns, como influenza, coxsackie e parvovírus.

Por ser subestimada clinicamente e pelo hábito do atleta de manter treinos mesmo durante sintomas infecciosos, a inflamação miocárdica pode passar despercebida e deixar sequelas permanentes. Após o surgimento da covid-19, a incidência de miocardite relacionada ao SARS-CoV-2 trouxe preocupação adicional, sobretudo em praticantes de atividades de alta intensidade.

**Figure:**
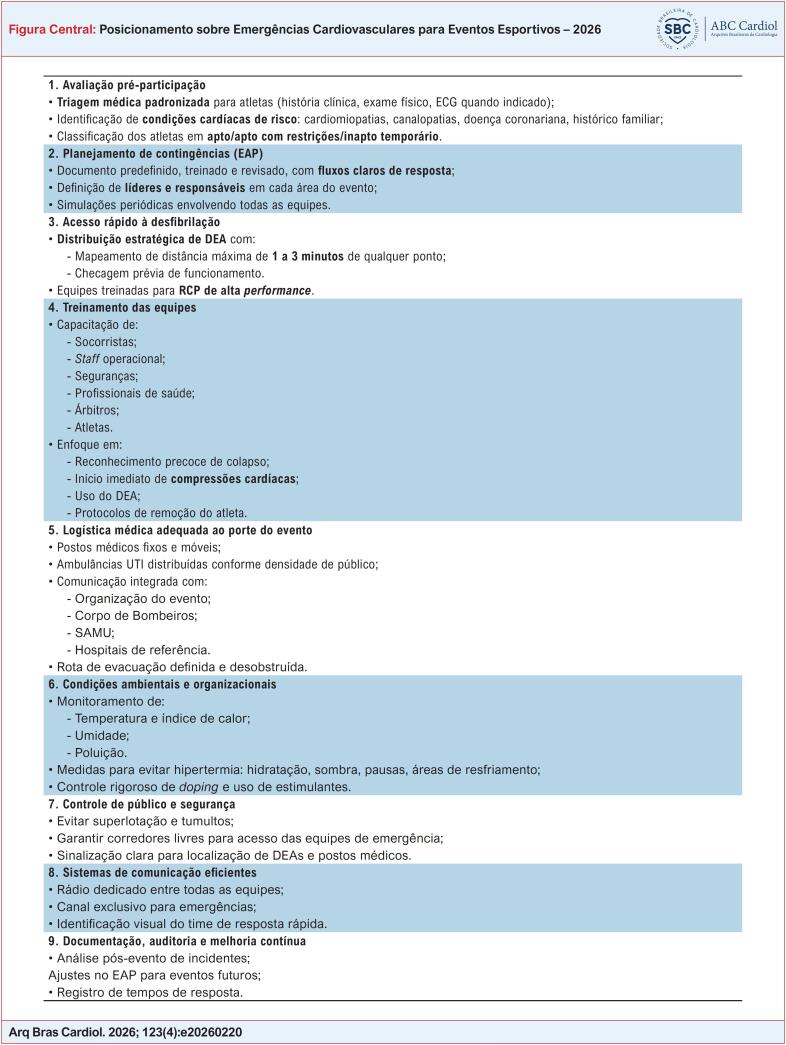
DEA: desfibrilador externo automático; EAP: emergency action plan; ECG: eletrocardiograma; RCP: reanimação cardiopulmonar; SAMU: Serviço de Atendimento Móvel de Urgência; UTI: unidade de terapia intensiva.

É importante ressaltar que nem toda morte súbita em atletas é de origem cardíaca. O uso disseminado de substâncias para a melhora de *performance* – como esteroides anabolizantes, estimulantes (anfetaminas, bebidas energéticas), hormônios peptídicos e drogas recreativas ilícitas, como a cocaína – tem sido relacionado ao aumento de doenças cardiovasculares adquiridas e ao risco de morte súbita em indivíduos jovens e de meia-idade.^10^ Outras causas não cardíacas também merecem atenção: hipertermia, distúrbios hidroeletrolíticos, trauma torácico, concussão, afogamento e síncopes relacionadas ao tipo de esporte.^11^

### 1.2. Epidemiologia e Incidência

A real incidência de morte súbita em atletas permanece imprecisa, em parte devido à heterogeneidade das fontes de dados disponíveis, que frequentemente incluem registros de mídia e relatórios de seguradoras, além da diversidade de populações estudadas. Estima-se que 56 a 80% dos casos de morte súbita em jovens atletas ocorram durante a prática esportiva, com a incidência variando entre 1 por 1 milhão e 1 por 5 mil atletas ao ano.^3,12,13^

Alguns grupos apresentam risco mais elevado, como atletas do sexo masculino, afrodescendentes e praticantes de basquetebol e futebol. Homens apresentam risco relativo maior que mulheres (3:1 a 9:1), enquanto afrodescendentes têm risco cerca de 3,2 vezes superior ao de brancos.

Em esportistas de basquetebol, a incidência anual de morte súbita chega a 1/9.000 em homens brancos e 1/5.300 em homens afrodescendentes.^3,12,14,15^

A avaliação pré-participação esportiva (APP) constitui a principal ferramenta preventiva da morte súbita no esporte, permitindo identificar indivíduos de risco e orientar condutas.

Além disso, a implementação de protocolos de emergência e a rápida resposta a eventos de colapso cardiovascular são determinantes para desfechos favoráveis.

### 1.3. Situação Atual: Lacunas entre Conhecimento e Prática

Apesar dos avanços significativos no conhecimento fisiopatológico da morte súbita no contexto esportivo e da disponibilidade de ferramentas diagnósticas e terapêuticas bem estabelecidas, persiste uma lacuna crítica entre a teoria e a prática em muitos cenários. Estudos evidenciam que paradas cardiorrespiratórias que ocorrem durante ou imediatamente após eventos esportivos ainda são frequentemente atendidas de forma inadequada, resultando em desfechos desfavoráveis que poderiam ser prevenidos.^16,17^

No Brasil, essa realidade assume características particulares. Enquanto alguns países desenvolvidos consolidaram estruturas robustas de resposta a emergências cardiovasculares em eventos esportivos, grande parte dos eventos realizados no território nacional carece de protocolos padronizados, de equipes adequadamente treinadas para reconhecer e responder prontamente à parada cardiorrespiratória, de desfibriladores externos automáticos (DEAs) estrategicamente posicionados e de capacitação em reanimação cardiopulmonar (RCP) entre organizadores e pessoal de segurança. Paralelamente, a APP médica, ferramenta crucial para identificar indivíduos em risco e prevenir a morte súbita ainda na sua origem, permanece inconsistentemente implementada em diferentes contextos e modalidades esportivas.^12^

A urgência dessa intervenção é amplificada por considerações fisiopatológicas críticas. A cada minuto de atraso no início da RCP, as chances de sobrevida reduzem-se aproximadamente entre 6 e 10%, representando uma diminuição progressiva na probabilidade de retorno da circulação espontânea (RCE).^16,18^ Ainda mais relevante, a melhor janela de oportunidade para intervenção ocorre nos primeiros minutos após o colapso, período em que os ritmos chocáveis – taquicardia ventricular (TV) e fibrilação ventricular (FV) – são mais frequentes e apresentam as maiores taxas de sucesso com desfibrilação.^19^ Passada essa janela crítica, há aumento progressivo da evolução para assistolia ou atividade elétrica sem pulso, dois ritmos não chocáveis associados a prognóstico substancialmente mais reservado e menores taxas de sobrevida.^18,19^

Diretrizes, como a da Sociedade Brasileira de Medicina do Esporte (SBME), publicada em 2005, estabeleceram recomendações robustas e bem fundamentadas para prevenção e manejo da morte súbita no esporte.^19^ No entanto, quase duas décadas depois, persiste uma distância significativa entre essas recomendações e sua implementação prática nos eventos esportivos brasileiros.

Uma análise sistemática da conformidade às recomendações da SBME 2005 em diferentes contextos esportivos brasileiros revela deficiências críticas em múltiplas dimensões (Tabela 1).^19^

**Tabela 1:** Recomendações para redução de morte súbita em eventos esportivos: implementação e impacto clínico

Dimensão	Nível de implementação	Impacto clínico
APP padronizada	30–40%	CRÍTICO – Falha na identificação de risco
Desfibriladores *in loco*	50–60%	CRÍTICO – Perda de janela de oportunidade
Treinamento em RCP	25–35%	CRÍTICO – Atraso na resposta
Protocolos	40–50%	ALTO – Heterogeneidade de condutas
Integração com SAMU	20–30%	ALTO – Demora em recursos avançados
Registro centralizado	10–15%	ALTO – Imprecisão epidemiológica
Legislação implementada	35–45%	MÉDIO – Falta de fiscalização

APP: avaliação pré-participação; RCP: reanimação cardiopulmonar; SAMU: Serviço de Atendimento Móvel de Urgência.

As deficiências mais críticas concentram-se em três pilares fundamentais:

- Prevenção primária através da APP adequada;
- Infraestrutura de resposta rápida com desfibriladores e equipes treinadas;
- Protocolos padronizados e integrados com sistemas de emergência.

Essa fragmentação de implementação resulta em variabilidade significativa de atendimento entre diferentes eventos e instituições, comprometendo a equidade de proteção aos atletas.

### 1.4. Justificativa e Chamada à Ação

Nesse contexto, iniciativas como este Posicionamento em emergências cardiovasculares em grandes eventos esportivos, liderado pelo Departamento de Ergometria, Reabilitação, Cardiologia do Esporte e Cardiologia Nuclear da Sociedade Brasileira de Cardiologia (SBC), ganham relevância estratégica ao proporem um marco referencial que integre tanto a prevenção quanto a resposta às emergências cardiovasculares.

A convergência de esforços entre sociedades médicas especializadas, associada ao envolvimento determinante de organizadoras de eventos, instituições esportivas e poder público, torna-se imperativa para transformar esse conhecimento em ação institucionalizada e salvar vidas.

O presente documento representa um avanço necessário na construção de políticas públicas e diretrizes operacionais que permitam não apenas a detecção precoce do risco individual, mas também a preparação adequada de todo o ecossistema de eventos esportivos, para responder efetivamente às emergências cardiovasculares, reduzindo a mortalidade evitável e garantindo que a prática desportiva seja segura para todos os participantes.

## 2. Avaliação Pré-Participação em Cardiologia do Esporte

A APP cardiológica tem como principal objetivo identificar doenças cardiovasculares potencialmente fatais que possam aumentar o risco de MSC e arritmias malignas durante a prática esportiva. Essa avaliação é recomendada para: atletas competitivos, praticantes de esportes recreativos, indivíduos que iniciam treinamento intenso.

A APP permite a detecção precoce de cardiopatias estruturais, canalopatias e outras condições de risco, sendo essencial para a segurança no esporte.^12,21,22^

### 2.1. Contexto Brasileiro

No Brasil, a APP é mais comum em clubes esportivos (como os de futebol) e em competições de corrida, trilhas e triatlo, que exigem liberação médica obrigatória. O Conselho Federal de Medicina (CFM) estabelece que a avaliação deve incluir:

- Anamnese detalhada;
- Exame físico completo;
- Exames complementares (quando indicado).

A SBC e a SBME recomendam protocolos adaptados à realidade nacional, considerando custo-efetividade, prevalência de doenças e infraestrutura disponível.^21^

O modelo brasileiro incorpora elementos do protocolo norte-americano (como os 14 pontos da anamnese e exame físico – Tabela 2) e do europeu, incluindo o eletrocardiograma (ECG) de repouso como exame obrigatório.

**Tabela 2:** Os 14 pontos da avaliação pré-participação

Categoria	Ponto	Critério
História clínica (8 pontos)	1	Dor/desconforto torácico durante exercício
	2	Síncope ou pré-síncope relacionada ao esforço
	3	Dispneia ou fadiga excessiva desproporcional
	4	História de sopro cardíaco
	5	Hipertensão arterial diagnosticada
	6	História familiar de morte súbita (< 50 anos)
	7	História familiar de cardiomiopatias, síndrome de Marfan ou arritmias graves
	8	Doença cardíaca congênita em parentes de primeiro grau
Exame físico (6 pontos)	9	Sopros patológicos
	10	Pulsos femorais diminuídos (suspeita de coarctação da aorta)
	11	Sinais de síndrome de Marfan (envergadura aumentada, deformidades torácicas)
	12	Medida da pressão arterial
	13	Bulhas anormais (B3, B4 ou galope)
	14	Arritmias detectadas no exame

A APP é, portanto, uma estratégia fundamental de prevenção, devendo ser conduzida por médicos especializados, com abordagem baseada em risco-benefício e alinhada às diretrizes nacionais e internacionais.

Um ponto importante na APP, para evitar, inclusive, exames desnecessários adicionais, é atentar-se para alterações eletrocardiográficas e de imagem que são relacionadas às adaptações ao treino inerentes ao atleta. Critérios internacionais para interpretação do ECG em atletas foram publicados e devem ser seguidos nas avaliações.^23,24^

### 2.2. Recomendações da Sociedade Brasileira de Cardiologia para a Avaliação Pré-Participação

1. Avaliação inicial
  - Anamnese detalhada (história pessoal e familiar);
  - Exame físico completo (com atenção a fatores de risco e sinais clínicos);
  - ECG (essencial para detectar alterações elétricas suspeitas).^23^
2. Exames complementares (conforme critério clínico)
  - Teste ergométrico (avaliação da resposta ao esforço, principalmente em adultos com risco moderado/alto);
  - Ecocardiograma (identificação de cardiomiopatias e alterações estruturais);
  - Exames laboratoriais (hemograma, glicose, lipidograma, função renal etc.);
  - Ressonância magnética cardíaca (em casos selecionados).^24^

### 2.3. Perfil e Padrão de Avaliação Recomendada

- < 35 anos, sem comorbidades: consulta médica + ECG;
- ≥ 35 anos ou com fatores de risco: consulta + ECG + exames laboratoriais + teste ergométrico ou outro exame funcional/anatômico de acordo com a probabilidade pré-teste de DAC;
- Atletas de elite: avaliação completa (ECG, ecocardiograma, teste funcional – ergométrico ou cardiopulmonar).

## 3. Checklist e Equipamentos Essenciais para o Atendimento de Emergências Cardiovasculares em Grandes Eventos Esportivos

O manejo de emergências cardiovasculares em grandes eventos esportivos requer um planejamento integrado, treinamento multiprofissional e infraestrutura adequada para garantir uma resposta rápida e eficaz frente à parada cardiorrespiratória (PCR) ou outras emergências cardíacas agudas.^25-27^

Um plano de ação de emergência (PAE) bem estruturado deve assegurar uma resposta rápida, a integração entre equipes médicas e a disponibilidade de equipamentos que permitam o atendimento imediato e eficaz. Esse plano é constituído por protocolos escritos e testados periodicamente e compõe o eixo central de qualquer estratégia de atendimento pré-hospitalar (APH) nesses cenários.^28^

As emergências cardiovasculares em campo podem ocorrer tanto em atletas aparentemente saudáveis – por diversas causas, como miocardiopatias, anomalias coronarianas, síndrome de QT longo ou cardiomiopatia arritmogênica do ventrículo direito e ventrículo esquerdo – quanto em membros da equipe técnica e no público.^26,29^

A literatura recomenda que o tempo entre o colapso e o primeiro choque para desfibrilação não ultrapasse 3 minutos.^29^ Assim, a disposição dos equipamentos e o treinamento das equipes determinam diretamente a taxa de sobrevivência pós-PCR, especialmente em ambientes de alta densidade populacional, como arenas, ginásios e pistas olímpicas.^28^

### 3.1. Estrutura do Plano de Equipamentos

O arsenal deve cobrir todo o espectro de suporte à vida:

- Suporte básico de vida (SBV) – voltado para primeiros respondedores e equipe auxiliar;
- Suporte avançado de vida (SAV) – executado por profissionais habilitados;
- Apoio logístico e trauma – destinado a imobilização, transporte e controle de lesões associadas.

Os equipamentos devem estar organizados em módulos (via aérea, ventilação, circulação, medicações e trauma) dentro de maletas ou mochilas identificadas, com *checklist* pré-evento obrigatório.^29^ Recomenda-se, também, o uso de protocolos impressos ou digitais para padronizar condutas de PCR, RCE e manejo do colapso no atleta.

O conjunto de materiais deve contemplar desde o SBV até o SAV em cardiologia, bem como dispositivos de comunicação, transporte e logística hospitalar.^27,29^ A lista deve ser adaptada conforme a modalidade esportiva, o porte do evento, o número de participantes e o tempo de resposta esperado.

Os equipamentos devem ser inspecionados antes de cada evento, testados quanto à funcionalidade e registrados em *checklist* digital ou impresso.^30^

### 3.2. Equipamentos de Suporte Básico de Vida (Tabela 3)

**Tabela 3:** Equipamentos de suporte básico de vida

Equipamento	Descrição e finalidade
DEA	Dispositivo indispensável para desfibrilação precoce. Deve estar posicionado de forma visível e acessível em até 3 minutos de qualquer ponto da arena.^29^ Recomendam-se baterias carregadas, pás sobressalentes e checagem antes do evento.
Bolsa-válvula-máscara ("ambu")	Essencial para ventilação manual em casos de apneia. Deve estar disponível em todos os pontos de atendimento.
Máscaras faciais e filtros HEPA	Utilizados para proteção contra contaminação cruzada e ventilação segura.
Cânulas orofaríngeas e nasofaríngeas (vários tamanhos)	Mantêm via aérea pérvia durante a RCP e facilitam uma ventilação eficaz.
Tesoura, lâmina de barbear e toalhas descartáveis	Permitem a rápida exposição torácica e a aplicação das pás do DEA.
Oxímetro de pulso portátil	Auxilia na monitorização inicial da saturação e frequência cardíaca durante a avaliação.

DEA: desfibrilador externo automático; HEPA: ar de partículas de alta eficiência (do inglês, high-efficiency particulate air); RCP: reanimação cardiopulmonar.

### 3.3. Equipamentos de Suporte Avançado de Vida (Tabela 4)

**Tabela 4:** Equipamentos de suporte avançado de vida

Equipamento	Descrição e aplicação
Monitor-desfibrilador manual e marcapasso transcutâneo	Permite análise de ritmo, registro de traçados e aplicação de desfibrilação manual por equipe treinada.^26^ O marcapasso transcutâneo com as pás adesivas é imprescindível para o manejo de bloqueios atrioventriculares.
Fonte de oxigênio portátil (≥ 5 L/min)	Deve incluir máscaras de reservatório, cânulas nasais e dispositivos de Venturi para suplementação adequada (com suas devidas conexões) além de cilindros de oxigênio portáteis.
Máscara laríngea e tubos endotraqueais (6,0–8,5 mm)	Alternativas para via aérea avançada em casos de falha na ventilação com ambu.
Aspirador portátil de secreções	Imprescindível para prevenir broncoaspiração durante manobras de RCP.
Acesso venoso periférico e intraósseo	Inclui cateteres, equipos e agulhas para infusão rápida.
Medicamentos de emergência	Devem estar disponíveis: adrenalina, amiodarona, atropina, dopamina, lidocaína em ampolas, nitroglicerina, aspirina, sulfato de magnésio 50% em ampolas, bicarbonato de sódio em ampolas, glicose hipertônica e bolsas de soro fisiológico 0,9%.^25,27^
Dispositivo de compressão torácica automática	Opcional, mas útil em RCPs prolongadas, especialmente em locais amplos.^28,29^

RCP: reanimação cardiopulmonar.

### 3.4. Comunicação e Logística Operacional

- Radiocomunicadores dedicados e sistema de comunicação fechado entre equipes médicas e de segurança;
- Protocolo de código de emergência padronizado, com designação de papéis claros;
- Mapas visuais indicando pontos de atendimento, rotas de ambulância e localização dos DEAs;
- Ambulância com unidade de terapia intensiva (UTI) móvel com equipe de SAV em cardiologia (ACLS, do inglês *advanced cardiac life support*) e espaço reservado para acesso rápido ao campo;^30,31^
- Identificação visual da equipe médica (coletes, crachás) e canal direto com o hospital de referência para coordenação pós-evento.

### 3.5. Equipamentos Específicos por Modalidade Esportiva (Tabela 5)

**Tabela 5:** Equipamentos específicos por modalidade esportiva

Modalidade	Equipamentos específicos	Racional clínico
Futebol	DEA, pranchas rígidas, colares cervicais, aspirador portátil e *kit* de via aérea avançada.	Alta incidência de colapsos por arritmias malignas e trauma torácico.
Basquete	DEA lateral à quadra, maleta de oxigênio, talas e imobilizadores.	Risco aumentado de PCR relacionada a cardiomiopatias e trauma torácico por impacto.
Atletismo	Postos móveis com DEA e oxigênio a cada 200–300 m.	Predomínio de síncope vasovagal, exaustão térmica e FV induzida por esforço.
Natação	DEA e via aérea próximos à piscina, prancha de flutuação, colar cervical impermeável, material para secar o tórax antes da aplicação das pás.	O atendimento deve considerar a remoção rápida da água, secagem do tórax para aplicar DEA e ventilação imediata.^26,29^
Artes marciais	DEA lateral ao tatame, *kit* de intubação, colar cervical, protetor facial e aspirador portátil.	Risco de trauma cervical, concussão e apneia reflexa pós-impacto.
Ciclismo/triatlo	Ambulância de prontidão, veículo de apoio (moto, bicicleta, lanchas ou caiaques) com DEA portátil e sistema de comunicação via rádio.	A extensão do percurso requer pontos de resposta distribuídos e mobilidade logística.^29,31^

DEA: desfibrilador externo automático; FV: fibrilação ventricular; PCR: parada cardiorrespiratória.

### 3.6. Procedimentos de Inspeção, Manutenção e Treinamento

A checagem prévia do equipamento, antes do início, por supervisão do coordenador médico do evento, deve seguir protocolo documentado, incluindo:

- Verificação de carga de baterias, testes dos desfibriladores, validade dos fármacos e integridade dos cabos e conexões, verificação dos cilindros de oxigênio;
- Simulações operacionais trimestrais com todas as equipes envolvidas (médicos, fisioterapeutas, árbitros e seguranças);
- *Checklist* digital ou impresso arquivado pela coordenação médica do evento;^30^
- Definição de rotas hospitalares e unidades de referência com suporte cardiológico avançado.

O treinamento periódico em RCP e uso de DEA para todos os profissionais e colaboradores envolvidos é considerado mandatório, conforme as recomendações da American Heart Association (AHA) e do European Resuscitation Council (ERC).^25,26,29^

Faz-se importante que as autoridades sanitárias locais (ex.: Vigilância Sanitária) façam auditorias no local para conformidade e adequações.

### 3.7. Considerações Finais

A eficiência na resposta a emergências cardiovasculares em grandes eventos esportivos depende da integração de três pilares principais:

1. Planejamento de atendimento estratégico (com PAE testado e atualizado);
2. Equipamentos adequados e funcionais;
3. Treinamento regular da equipe multiprofissional.

A implantação de *checklists* específicos por modalidade e o monitoramento da prontidão logística reduzem significativamente o tempo até a desfibrilação e melhoram os desfechos de sobrevida. A equipe médica deve estar apta não apenas ao SAV, mas também ao manejo do trauma, da hipertermia e da anafilaxia, condições comuns nesses contextos. A inspeção regular dos equipamentos e o treinamento interdisciplinar são pilares inegociáveis para garantir a segurança de atletas e espectadores.

A prevenção de mortes súbitas evitáveis em atletas e público requer, portanto, um modelo sistematizado de resposta, com protocolos baseados em evidências e revisões contínuas de qualidade.^27,30,32^

## 4. Componentes da Equipe, Formação e Capacitação

Em grandes eventos esportivos, é necessário que haja um diretor ou coordenador médico que será o responsável pela elaboração de um plano de ação para o atendimento de emergências cardiovasculares e pela elaboração de protocolos atualizados e que fará a interface entre a equipe que dará o primeiro atendimento e o serviço de emergência para onde o paciente eventualmente será levado.

A SBC dispõe de cursos em vários níveis para o suporte de vida, desde cursos para população leiga (treinamento de emergências cardiovasculares avançado, TECA L) e para profissionais de saúde nas modalidades básico (TECA B) e avançado (TECA A).^33-36^

O diretor médico obrigatoriamente deverá ter registro ativo no Conselho Regional de Medicina (CRM), ter experiência em atendimentos de emergência e de gestão de serviços médicos em eventos. Além da experiência, deverá ser capacitado em atendimento avançado de emergências cardiovasculares (TECA A).^35^

O número de profissionais participantes da equipe médica e envolvidos no atendimento dependerá do número total de atletas participantes e deverá respeitar a legislação local vigente. A equipe de atendimento (incluindo viatura de ambulância ou nos postos de atendimento) deverá ser composta, preferencialmente, por um médico, um enfermeiro e um técnico de enfermagem, além do condutor (para as ambulâncias).^33-36^

O médico deve ter registro ativo no CRM e, idealmente, formação/experiência em uma das seguintes áreas: emergência, terapia intensiva, medicina do esporte, ortopedia ou cardiologia. Ele também deverá, obrigatoriamente, ser capacitado em atendimento avançado de emergências cardiovasculares (ex.: TECA A).^35^

O enfermeiro também deverá ter formação/experiência em atendimentos de emergência e ser capacitado em atendimento avançado de emergências cardiovasculares (ex.: TECA A). Já o técnico de enfermagem, além de experiência em emergências, deverá ser capacitado em atendimento básico de emergências cardiovasculares (TECA B). Os condutores de ambulância devem, idealmente, possuir treinamento para emergências cardiovasculares (TECA L). A depender da logística, tipo de evento esportivo e do plano de atendimento, socorristas ou bombeiros civis capacitados em primeiros socorros e em SBV devem ser agregados à equipe de resposta rápida.^33-36^

Em eventos envolvendo clubes ou equipes profissionais, com equipe médica própria para o atendimento dos atletas, o médico do clube deverá ter registro ativo no CRM, formação em emergência, cardiologia, ortopedia ou medicina do esporte e deverá, obrigatoriamente, ser capacitado em atendimento básico de emergências cardiovasculares (TECA B). Além disso o PAE, a equipe de atendimento e a localização dos desfibriladores (sejam automáticos ou não) devem estar estabelecidos *a priori*. Idealmente, antes de qualquer evento, deve-se estabelecer a função desempenhada por cada membro no caso da ocorrência de uma emergência.

É fortemente recomendado que membros de equipe técnica, e mesmo os atletas, tenham capacitação em SBV (mínimo TECA L, desejável TECA B), uma vez que, em muitas situações, eles podem ser os primeiros a presenciar um colapso e devem ser capazes de identificar a ocorrência como uma potencial parada cardíaca, iniciar manobras de RCP e solicitar ajuda e o DEA.

## 5. Equipamentos, Localização e Logística em Eventos Esportivos

### 5.1. Introdução

Os grandes eventos esportivos, sejam profissionais ou recreativos, reúnem um elevado número de atletas e espectadores em ambientes de alta demanda emocional, fisiológica e logística. Essa combinação cria um cenário propício ao aumento do risco de emergências cardiovasculares, em especial, a PCR.

Estudos recentes demonstram que, embora a incidência de parada cardíaca em arenas esportivas seja relativamente baixa, a probabilidade de sobrevida depende quase exclusivamente da pronta disponibilidade de DEAs, pessoas treinadas e da organização logística do atendimento imediato. O tempo entre o colapso e a desfibrilação é o principal determinante da sobrevivência, devendo, idealmente, ser inferior a 3 minutos. Cada atraso de 5 minutos no RCE em pacientes que sofrem parada cardiorrespiratória extra-hospitalar está associado a um aumento de 38% no risco de morte.^37-39^

**Figura 1:**
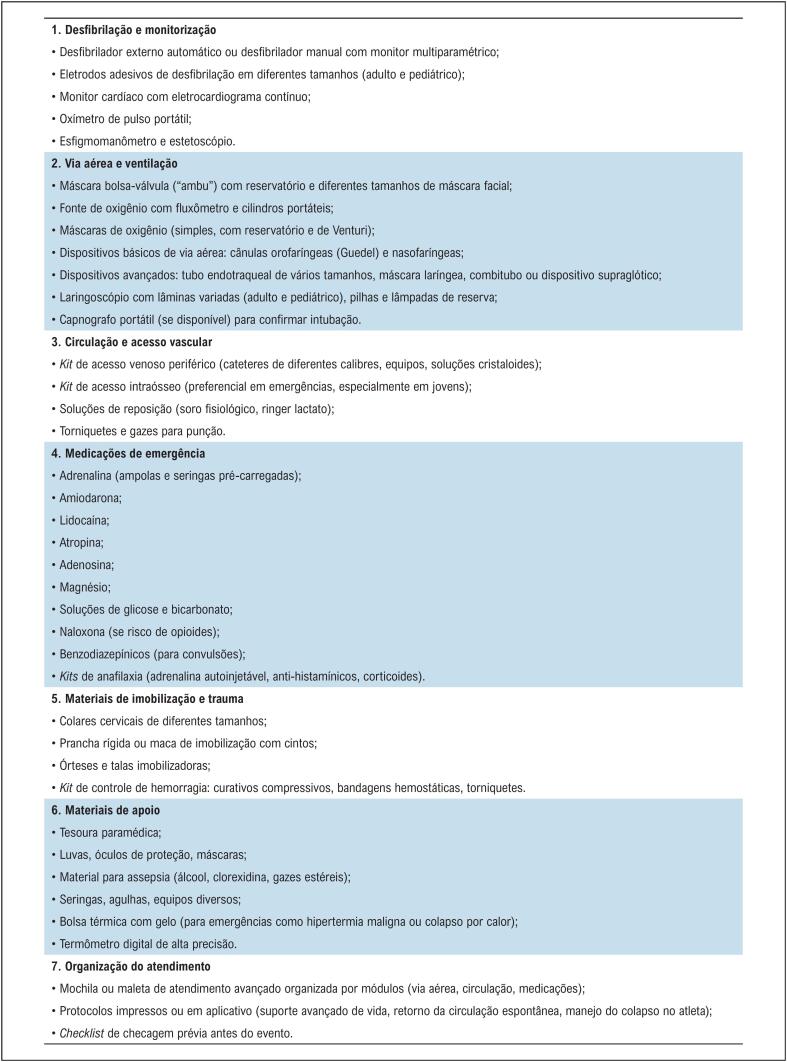
Lista dos equipamentos e medicamentos necessários em caso de emergências médicas em grandes eventos esportivos.

Diante disso, o posicionamento da SBC sobre emergências cardiovasculares em grandes eventos esportivos enfatiza três pilares estruturantes: equipamentos, treinamento/capacitação, localização estratégica e logística integrada, todos embasados em evidências nacionais e internacionais e na experiência de programas de segurança cardiovascular em arenas europeias e norte-americanas (Figura 1).

### 5.2. Equipamentos Essenciais

#### 5.2.1. Desfibriladores Externos Automáticos

Cada arena ou espaço do evento deve dispor de um mapa detalhado, contendo as rotas de evacuação, a localização de DEAs, das salas médicas e dos acessos de ambulância. Esse documento deve integrar o plano operacional do evento, servindo como referência para todas as equipes envolvidas no atendimento emergencial.

A distribuição estratégica de DEA é a medida mais eficaz para reduzir a mortalidade por PCR em eventos esportivos. A European Society of Cardiology (ESC) recomenda um tempo máximo de 3 minutos entre o colapso e a aplicação do primeiro choque, o que requer um número adequado de aparelhos posicionados de forma equidistante dentro do estádio ou no percurso, que é calculado pela área e tempo médio de deslocamento. As diretrizes europeias sugerem: 1 a 2 DEAs para arenas com até 10.000 pessoas; 4 DEAs para 10.000 a 50.000 pessoas; 8 ou mais para arenas acima de 50.000 pessoas.^40^

Os DEAs devem estar devidamente sinalizados, com verificação prévia do funcionamento, incluindo bateria e *pads* (eletrodos adesivos), e posicionados próximos aos acessos de emergência e às áreas de maior concentração de pessoas, como arquibancadas centrais, áreas VIP, corredores de circulação e zonas de imprensa.

#### 5.2.2. Material de Suporte Avançado

Além do DEA, a estrutura deve incluir (vide Seção 3):

- Oxigênio suplementar e "ambu";
- Monitor cardíaco e oxímetro;
- *Kit* de via aérea (cânulas, laringoscópio, tubo orotraqueal);
- Medicamentos de SAV (adrenalina, amiodarona, lidocaína, solução salina etc.);
- Macas, pranchas e cadeiras de transporte;
- *Kits* de resfriamento (tratamento de hipertermia).

#### 5.2.3. Recursos Complementares

Equipamentos de comunicação (rádios, sistema central de coordenação), acesso à ambulância e salas médicas equipadas compõem a estrutura mínima necessária para garantir a segurança em arenas de grande porte.

##### 5.3. Localização e Infraestrutura de Atendimento

#### 5.3.1. Mapeamento Interno

Cada arena ou local de prova deve dispor de um mapa detalhado com as rotas de evacuação, a localização dos DEAs, dos postos médicos e dos acessos de ambulância. Esse documento deve compor o plano de ação médica (PAM), o qual deve ser revisado e amplamente divulgado antes de cada evento. Os pontos críticos incluem:

- Portões principais e de serviço, com espaço reservado para ambulâncias;
- Áreas de difícil acesso (arquibancadas superiores, zonas de torcida organizada);
- Setores de atletas e arbitragem, com entrada direta à unidade médica.

#### 5.3.2. Salas Médicas e Postos de Atendimento

Deve haver pelo menos uma sala médica a cada 10.000 espectadores, equipada para suporte avançado e com acesso direto à ambulância. Em arenas ou eventos com mais de 50.000 pessoas, recomenda-se a instalação de salas adicionais setoriais. Essas unidades devem ter visibilidade e sinalização adequadas, integradas ao sistema de comunicação e aos protocolos de atendimento do Serviço de Atendimento Móvel de Urgência (SAMU) local.

#### 5.3.3. Transporte Interno e Externo

O deslocamento do paciente até o hospital de referência deve ocorrer em menos de 5 minutos. No interior da arena, é essencial garantir rotas de acesso desobstruídas, bem como a disponibilidade de veículos elétricos ou macas motorizadas para transporte em áreas de difícil alcance. Externamente, recomenda-se a presença de no mínimo uma ambulância para cada 10.000 pessoas, equipada com SAV (UTI móvel) e integrada à coordenação direta com o hospital de referência.

A organização do evento deve garantir um serviço de APH próprio, com ambulâncias posicionadas no local e capacidade de realizar a estabilização inicial e o transporte imediato para o serviço de referência. Esse sistema pode atuar de forma independente do APH público (como SAMU ou Corpo de Bombeiros), embora possa ser acionado e integrado ao apoio dessas instituições em situações de maior complexidade, como eventos com múltiplas vítimas.

### 5.4. Logística Operacional

#### 5.4.1. Plano de Ação de Emergência

O PAM deve incluir:

- Nome e função do diretor médico do evento, responsável por todas as decisões clínicas;
- Descrição da equipe (médicos, enfermeiros, técnicos de enfermagem, socorristas e voluntários);
- Fluxograma de acionamento e comunicação;
- Localização de equipamentos e salas médicas;
- Protocolos de atendimento e triagem;
- Comunicação com serviços externos (SAMU, bombeiros, hospitais de referência);
- Procedimentos de documentação e relatório de eventos adversos.

O plano deve ser testado e simulado anualmente, incluindo treinamento prático com todas as equipes envolvidas.

#### 5.4.2. Equipe e Treinamento

A European Association of Cardiovascular Prevention and Rehabilitation (EACPR), seção da Cardiologia do Esporte, recomenda a presença mínima de:

- 2 médicos por 50.000 espectadores;
- 1 enfermeiro por 10.000 pessoas;
- 2 técnicos de emergência por 10.000 pessoas;
- Pelo menos 50% dos voluntários treinados em reanimação e uso de DEA.^40^

Além das recomendações presentes neste documento, devem também ser observadas as legislações vigentes relativas ao tema na localidade em que se dará o evento.

Todos os membros das equipes de atendimento devem portar identificação visível, rádios de comunicação e uniforme padronizado. O treinamento deve seguir os protocolos do ERC e incluir simulações realistas de PCR, com análise de desempenho e *debriefing* após cada exercício ou ocorrência real.^40^

#### 5.4.3. Comunicação e Central de Comando

O centro de operações deve atuar como núcleo de coordenação médica, dispondo de canal exclusivo de rádio, linha direta com o hospital de referência e monitoramento em tempo real de todas as ocorrências. A realização de testes prévios de cobertura e de redundância dos sistemas de comunicação é obrigatória para assegurar a eficiência operacional e o tempo de resposta ideal durante o evento.

### 5.5. Impacto dos Equipamentos e da Logística sobre a Sobrevida

Evidências robustas sustentam que a presença de equipes treinadas e de DEAs reduz drasticamente a mortalidade:

- Em arenas com DEA e resposta ≤ 3 min, a taxa de sobrevivência alcança 60 a 70%, com recuperação neurológica completa na maioria dos casos. Em locais sem DEA ou plano estruturado, a sobrevivência raramente ultrapassa 5%;^26,40,41^
- No estudo francês de Marijon et al., a sobrevivência de paradas cardíacas em instalações esportivas foi de 22,8%, contra 8% fora delas, diferença atribuída à presença de testemunhas, reanimação precoce e melhor logística;^42^
- Casos documentados, como no estádio de Alkmaar (Holanda), mostraram 100% de sobrevida em quatro paradas cardíacas consecutivas, todas revertidas com DEA e transporte rápido;^41^
- Apesar de o risco de parada cardíaca durante maratonas nos EUA manter-se estável ao longo do tempo, a mortalidade reduziu quase pela metade desde 2010. Esse avanço reflete a melhoria na resposta imediata às emergências, especialmente pela aplicação precoce da RCP por testemunhas e pelo uso rápido de DEAs, medidas que comprovadamente aumentam as chances de sobrevivência em corridas de longa distância.^43^

### 5.6. Considerações Operacionais para o Contexto Brasileiro

Nos grandes eventos nacionais, como partidas de futebol, corridas de rua, triatlos, maratonas e eventos híbridos, a implementação das diretrizes internacionais deve ser:

- Inclusão do SAMU e do Corpo de Bombeiros nos protocolos prévios;
- Definição de hospital de referência com capacidade cardiológica 24 h;
- Integração com autoridades esportivas e policiais para controle de fluxos e segurança;
- Previsão de condições climáticas extremas, com planos específicos para calor intenso e desidratação;
- Treinamento anual de médicos de campo, fisiologistas e preparadores físicos para o reconhecimento precoce de sinais de colapso cardiovascular.

### 5.7. Conclusão

A organização de emergências cardiovasculares em grandes eventos esportivos exige muito mais do que a simples disponibilidade de um desfibrilador: requer sistema, equipe e protocolo bem definidos. Equipamentos adequados, posicionamento estratégico e logística eficiente compõem uma cadeia de sobrevivência integrada, em que cada minuto é determinante. Modelos propostos pela EACPR e pela AHA demonstram que a preparação salva vidas e que cada arena ou competição deve ser planejada como um microambiente hospitalar, com estrutura própria e comunicação plena com o sistema de urgência.

**Figura 2:**
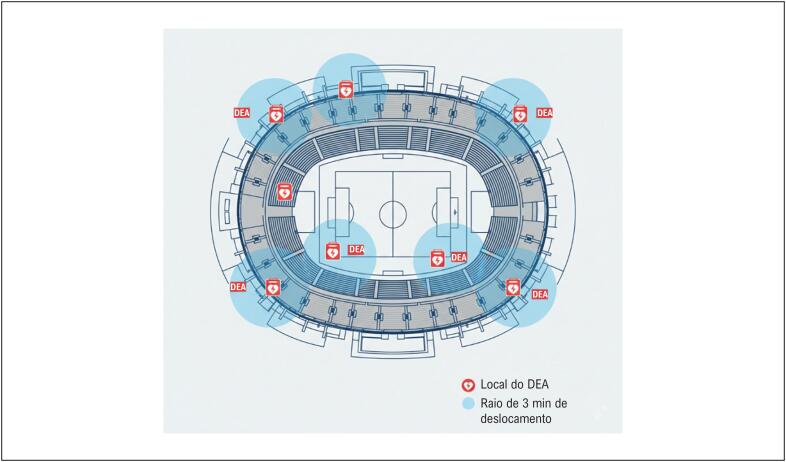
Disposição ideal dos desfibriladores externos automáticos (DEA) em um estádio de grande porte

A Figura 2 representa a disposição ideal dos DEAs em um estádio de grande porte, garantindo a cobertura completa em até 3 minutos de deslocamento a partir de qualquer ponto das arquibancadas ou áreas técnicas. A localização equidistante dos equipamentos assegura tempo de resposta compatível com a cadeia de sobrevivência e otimiza as chances de reversão da PCR.

**Figura 3:**
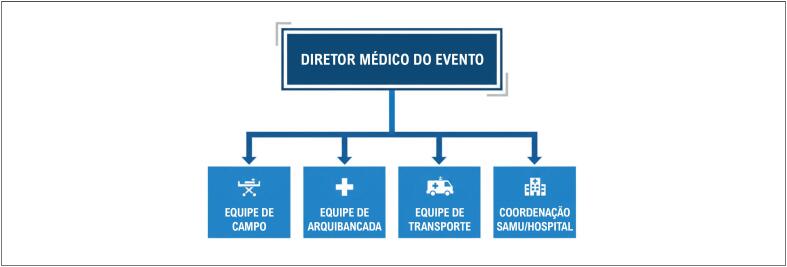
Organograma proposto para a equipe médica em eventos esportivos. SAMU: Serviço de Atendimento Móvel de Urgência.

A Figura 3 e a Tabela 6 apresentam as recomendações de equipamento e pessoal da EACPR.

**Tabela 6:** Recomendações de equipamentos e pessoal

Capacidade da arena	DEAs	Médicos	Enfermeiros	Técnicos	Ambulâncias
< 10.000	1–2	1	1	2	0–1
10–50.000	4	2	1–5	2–10	1–2
> 50.000	8+	2–4	> 5	> 10	> 2

DEAs: desfibriladores externos automáticos.

**Figura 4:**
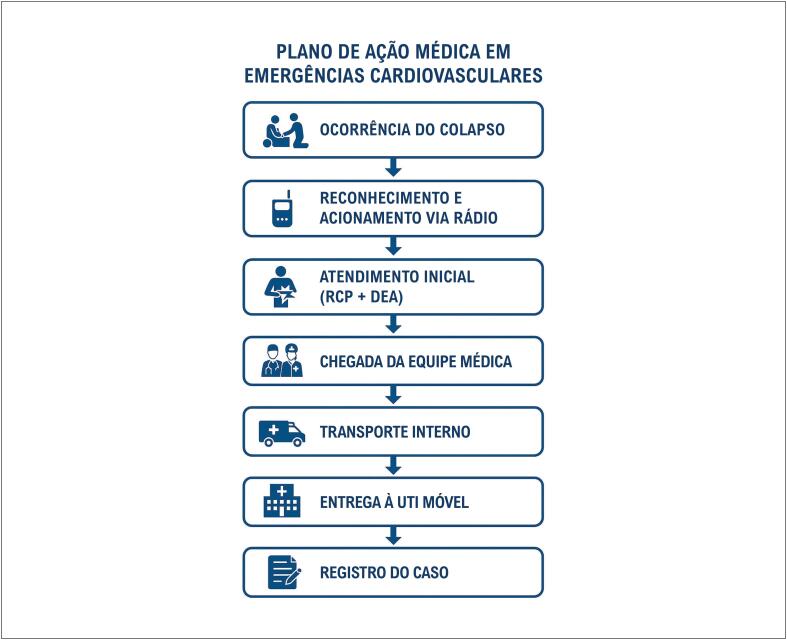
Fluxograma do plano de ação médica em emergências cardiovasculares. DEA: desfibrilador externo automático; RCP: reanimação cardiopulmonar; UTI: unidade de terapia intensiva.

A Figura 4 apresenta a sequência padronizada de resposta médica a colapsos cardiovasculares em arenas esportivas, integrando reconhecimento imediato, RCP com uso do DEA, chegada da equipe médica, transporte interno, entrega à UTI móvel e comunicação final com o hospital, seguida do registro do caso.

## 6. Logística de Atendimento, Evacuação e Transporte

A realização de grandes eventos esportivos representa um desafio de planejamento assistencial, segurança pública e emergências médicas. Classificado como *"mass gathering medicine"*, é uma área interdisciplinar dedicada ao estudo das implicações de saúde em eventos que reúnem grande número de pessoas em um mesmo espaço e tempo, podendo gerar demandas assistenciais que excedem a capacidade do sistema local.^44^

No contexto esportivo, eventos como maratonas, triatlos, jogos olímpicos, campeonatos mundiais e grandes torneios de futebol, apresentam maior complexidade logística. Esses eventos envolvem não apenas atletas de diferentes idades e níveis de preparo, mas também espectadores, voluntários e profissionais de apoio. As emergências cardiovasculares representam uma fração menor de ocorrências médicas, mas exigem resposta imediata e coordenada.^45^ Essa resposta deve ter um sistema integrado, baseado em planejamento prévio, infraestrutura adequada, equipes multidisciplinares, protocolos claros de triagem, comunicação eficiente e integração hospitalar, objetivando o menor intervalo possível entre o colapso clínico e a intervenção definitiva, respeitando o princípio do "tempo-dependente".

Médicos do esporte, cardiologistas, ortopedistas, emergencistas/intensivistas desempenham papel central nesse processo, desde o planejamento prévio de atendimento e da infraestrutura médica necessária, até o atendimento imediato, estabilização de casos críticos ou a tomada de decisão sobre evacuação e transporte.

### 6.1. Planejamento e Infraestrutura Médica

#### 6.1.1. Avaliação de Risco Pré-Evento

O planejamento de atendimento deve começar meses antes da competição, com base em uma avaliação de risco abrangente, incluindo:

- Perfil epidemiológico dos atletas (faixa etária, prevalência de comorbidades, nível competitivo);
- Condições ambientais (clima, umidade, altitude, poluição atmosférica);
- Histórico de incidentes em eventos anteriores semelhantes;
- Capacidade dos serviços locais de saúde (hospitais, SAMU, bombeiros).

Um estudo qualitativo na Maratona de Atenas demonstrou que a transferência interorganizacional de conhecimento entre saúde, segurança e gestores do evento foi determinante para alinhar protocolos de triagem, evacuação e integração hospitalar.^46^ Essa coordenação interinstitucional é atualmente considerada um fator crítico de sucesso em *mass gathering medicine.*

#### 6.1.2. Estrutura Assistencial

A infraestrutura de atendimento deve contemplar uma combinação de postos médicos fixos e equipes móveis, de modo a garantir o acesso rápido às vítimas em qualquer ponto do evento. As experiências mais recentes sugerem a adoção de postos médicos avançados (PMAs) estrategicamente distribuídos, capazes de realizar triagem, estabilização clínica inicial, SAV e decisão de evacuação.^45^

Os PMAs devem ser dimensionados para atender tanto emergências críticas quanto demandas de baixa complexidade, prevenindo a sobrecarga das ambulâncias e do sistema hospitalar.

#### 6.1.3. Comunicação e Comando

O centro de comando médico (CCM) deve integrar todas as informações sobre ocorrências, recursos disponíveis, localização das equipes e rotas de evacuação. Falhas de comunicação estão entre as principais causas de atrasos assistenciais em eventos de massa.^44,46^

Ensaios práticos com simulações realísticas de cenários críticos – como parada cardíaca no percurso – aumentam a eficiência da equipe e reduzem significativamente o tempo de resposta. Eventos como os Jogos Olímpicos de Tóquio 2020 e de Pequim 2022 incluíram simulações obrigatórias para todos os profissionais envolvidos.^47^

#### 6.1.4. Operação no Evento

A fase operacional de grandes eventos esportivos é caracterizada pela necessidade de resposta tempo-dependente a emergências médicas em meio a um ambiente dinâmico, com múltiplos fatores envolvidos. A complexidade advém da dispersão geográfica dos atletas, da presença de público numeroso e da limitação de acesso rápido a determinados locais do percurso ou das arenas esportivas.

#### 6.1.5. Postos Médicos Avançados

Os PMAs constituem o eixo central da logística assistencial. Em maratonas, recomenda-se instalar PMAs principais próximos à largada e à chegada, complementados por postos satélites ao longo do percurso em áreas de maior risco (subidas longas, pontos de calor, trechos de maior concentração de público). Já em eventos de estádio, os PMAs devem estar localizados atrás das arquibancadas principais, próximos a acessos para ambulâncias.^45,48^

#### 6.1.6. Equipes Móveis

As equipes móveis desempenham papel crucial para redução do tempo-resposta, e diversas modalidades podem ser incluídas:^49^

- Ciclomédicos: em maratonas e triatlos, com mochilas contendo DEA, oxímetro, materiais de via aérea e analgésicos simples;
- Motolâncias: utilizadas em vias urbanas congestionadas;
- Lanchas ou caiaques: utilizadas em provas em mar aberto ou lagoas;
- Equipes pedestres: em arenas esportivas;
- Veículos 4x4: em terrenos irregulares, como provas de aventura ou motocross;
- Drones para transporte de DEA: estudos apontam o uso experimental de drones com DEA em ambientes ao ar livre, com potencial para reduzir o tempo até a desfibrilação em cenários simulados.^50^

Um estudo japonês de emergências em estádios esportivos entre 2015 e 2019 concluiu que os incidentes clinicamente graves e potencialmente fatais ocorrem frequentemente em locais como piscinas, sanitários e áreas de assento de espectadores. Esses resultados enfatizam a necessidade de sistemas de emergência médica abrangentes, incluindo o posicionamento estratégico de DEAs, para atender não apenas atletas, mas também espectadores e funcionários.^51^

#### 6.1.7. Comunicação e Centro de Comando

A operação deve estar centralizada em um CCM, que integra comunicação entre PMAs, equipes móveis, ambulâncias e hospitais de referência. Protocolos padronizados são recomendados para assegurar a clareza nas informações transmitidas. A comunicação efetiva entre organizadores, serviços de saúde e segurança é determinante para o manejo de incidentes, reduzindo o tempo de triagem e evacuação.^46^

### 6.2. Atendimento Médico e Triagem

#### 6.2.1. Logística Durante o Atendimento Médico

Uma PCR deve ser reconhecida e atendida em até 3 minutos, com uso imediato de DEA. Em uma análise de vídeos de casos de PCR durante atividades esportivas, todos os atletas que receberam desfibrilação em até 3 minutos sobreviveram (100%).^53^

Procedimentos de SAV podem ser iniciados caso haja equipe treinada no local, mas isso não deve atrasar ou interromper as compressões torácicas, o uso do desfibrilador ou a decisão de transportar o atleta. A administração de epinefrina 1 mg e de amiodarona 300 mg por via intravenosa (IV) ou intraóssea (IO) deve ser realizada após o terceiro choque, se o atleta permanecer sem resposta antes da remoção do campo de jogo. Se o ritmo mudar de chocável para não chocável por mais de um ciclo de RCP, deve-se considerar a transferência imediata para um hospital. Porém, se houver dificuldades para mover o paciente, deve-se minimizar a interrupção da RCP, e continuar *in situ* (mesmo após três choques) pode ser uma opção melhor.

#### 6.2.2. Registro e Indicadores de Qualidade

Todos os atendimentos devem ser registrados em prontuário padronizado, incluindo horário do início dos sintomas, tempo até o primeiro contato, intervenções realizadas e desfecho. Esses dados permitem o cálculo de indicadores como tempo médio até o DEA e taxa de transporte hospitalar, fundamentais para a auditoria pós-evento.

### 6.3. Evacuação Médica

#### 6.3.1. Critérios de Evacuação

A decisão de evacuação médica em eventos esportivos deve considerar tanto a gravidade clínica quanto a possibilidade de estabilização inicial no local e a disponibilidade de recursos hospitalares especializados. Diretrizes internacionais de triagem no campo recomendam o transporte prioritário de pacientes com critérios de maior gravidade, como:^55,56^

- Alteração de consciência com escala de coma de Glasgow ≤ 13;
- Instabilidade hemodinâmica ou choque não responsivo a medidas iniciais;
- Necessidade de intervenção definitiva em hospital de referência (ex.: Hemodinâmica para infarto agudo do miocárdio).

#### 6.3.2. Rotas de Evacuação

A boa interação com a equipe de cuidados pós-PCR é fundamental. O tempo de deslocamento até o local predefinido deve ser o mais curto possível. As rotas dedicadas de evacuação devem ser planejadas previamente, evitando cruzamento com atletas e áreas de público denso. É recomendada a criação de pontos de interceptação ao longo do percurso para o acesso rápido de ambulâncias. No Japão, um estudo recente destaca que a falta de rotas definidas foi um dos principais fatores de atraso em eventos esportivos de massa.^51^

#### 6.3.3. Integração com Hospitais de Referência

Hospitais de referência devem ser previamente notificados sobre o evento, com definição de quais unidades receberão casos cardiovasculares, traumáticos e pediátricos. A pré-notificação hospitalar estruturada, com informação da evolução do atendimento, é fundamental para preparar a equipe de destino (que deve ser familiarizada e experiente com os protocolos) e reduzir tempo de intervenção.^46^

#### 6.3.4. Transporte Seguro

O transporte deve ocorrer apenas após a estabilização inicial das funções vitais, com exceção de cenários em que o tratamento definitivo só pode ser realizado no hospital (ex.: hemodinâmica para infarto com supra de ST). As diretrizes atuais não recomendam o transporte da vítima durante a RCP, porque sua eficiência diminui e aumenta-se o risco de lesões aos socorristas realizando manobras dentro do veículo em movimento, sendo o ideal o transporte após a recuperação do ritmo organizado. A RCP em andamento só é eficaz e segura se houver dispositivos mecânicos de compressão ou se a distância for mínima. O transporte manual em ambulância em movimento reduz a qualidade da RCP e expõe a equipe a riscos desnecessários. Em situações de recorrência de PCR no transporte, as medidas de ressuscitação devem continuar, se necessário, por ambulância aérea ou rodoviária durante o transporte, sendo realizada por pessoal treinado durante a movimentação ou, preferencialmente, com dispositivos automatizados de compressão torácica externa.

Em casos de suspeita de hipertermia grave (> 40 ºC), o resfriamento imediato (imersão/toalhas) precede o transporte ("*Cool first, transport second*").

#### 6.3.5. Monitorização Durante o Transporte

- ECG contínuo em pacientes cardiovasculares;
- Oximetria de pulso, temperatura e pressão arterial seriada em todos os transportes;
- Capnografia em pacientes ventilados;
- Comunicação contínua com o hospital receptor.

Após a PCR, pode ocorrer hipotermia leve no paciente comatoso, e o aquecimento ativo não deve ser realizado, realizando-se a manutenção de hipotermia com alvo de temperatura entre 32 e 36 °C por 12 a 24 horas e o controle ativo da prevenção da febre (temperatura ≤ 37,5 °C) por 72 horas. Dispositivos de refrigeração com *feedback* de controle de temperatura são preferíveis à realização de resfriamento pré-hospitalar com fluidos frios IVs, devido ao risco de nova parada e edema pulmonar.

### 6.4. Aspectos Legais e de Segurança

É necessário documentar o consentimento ou a recusa de transporte, assegurar a confidencialidade dos dados clínicos (Lei Geral de Proteção de Dados Pessoais) e garantir a segurança operacional da rota, evitando interferência de público ou tráfego urbano.^48^

Um dos elementos mais relevantes para a melhoria contínua da logística assistencial é a transferência de conhecimento entre organizadores, equipes médicas, serviços de emergência e hospitais de referência.

Um estudo recente sobre a Maratona de Atenas evidenciou que a ausência de protocolos claros compartilhados entre as instituições aumentava o risco de duplicação de esforços, falhas de comunicação e atrasos em evacuações críticas. A implementação de reuniões interinstitucionais prévias, simulações conjuntas e reuniões diárias durante o evento foi associada a maior eficiência operacional e redução de tempo-resposta.^46^

Outro exemplo relevante é o modelo de hospital de campo utilizado na Ryder Cup 2023, no qual médicos do esporte, cardiologistas, ortopedistas e emergencistas atuaram lado a lado em um sistema modular de atendimento. Esse formato permitiu a coleta de dados em tempo real, posteriormente utilizados para avaliar pontos de melhoria, como reposicionamento de ambulâncias, ajuste de estoques de medicamentos e treinamento adicional em hipertermia.^45^

Auditorias pós-evento, acompanhadas de relatórios de desfecho clínico e indicadores de processo [tempo até desfibrilação, taxa de resfriamento em insolação por esforço físico (*exertional heat stroke*) iniciado no local, proporção de casos estabilizados *in loco* vs. transferidos] são ferramentas indispensáveis para retroalimentar o planejamento de futuras competições.^44^

### 6.5. Considerações por Ambiente

**Figura 5:**
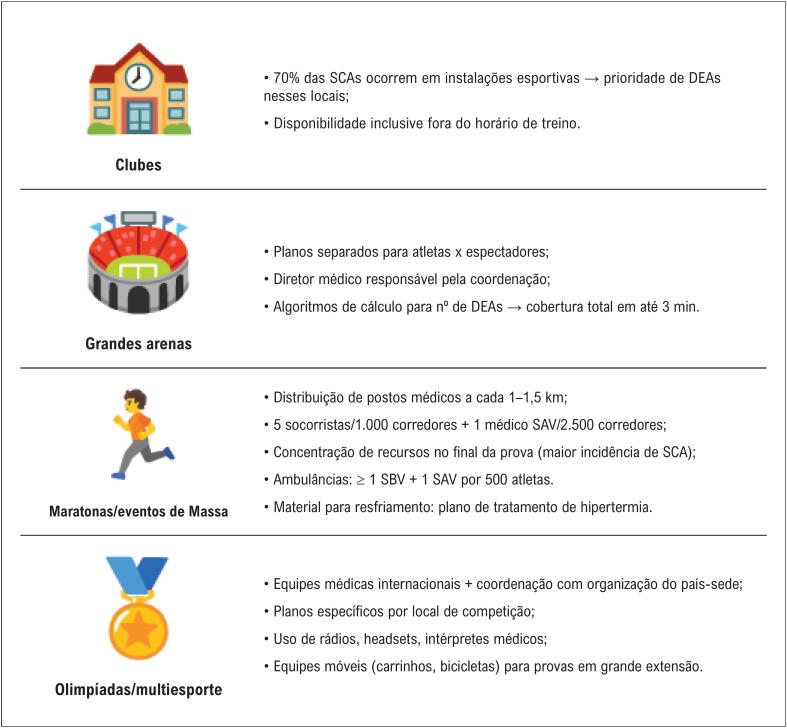
Especificidades por ambiente. DEA: desfibrilador externo automático; SAV: suporte avançado de vida; SBV: suporte básico de vida; SCA: parada cardíaca súbita (do inglês, sudden cardiac arrest).

- Clubes: priorizar o DEA em áreas esportivas; acesso inclusive fora do horário treino;
- Grandes arenas: planos separados para atletas e espectadores; diretor médico deve coordenar recursos;
- Maratonas e eventos de massa: até 5 socorristas por 1.000 atletas e pelo menos 1 médico de suporte avançado a cada 2.500 corredores; postos de socorro a cada 1,5 km aproximadamente; ambulâncias posicionadas **principalmente na segunda metade do percurso**;
- Olimpíadas: logística de múltiplos locais; necessidade de equipes móveis e intérpretes médicos; treinamento conjunto para uniformizar resposta (Figura 5).

## 7. Protocolos de Atendimento Básico e Avançado (a Cadeia de Sobrevivência) Adaptados ao Esporte

A PCR em atletas, embora rara, é um evento catastrófico que exige uma resposta imediata, coordenada e eficiente. A adaptação da "Cadeia de Sobrevivência" da AHA ao ambiente esportivo é crucial para melhorar os desfechos.^25,56-59^

### 7.1. Reconhecimento Imediato e Ativação do Serviço de Emergência

- Adaptação esportiva: dificuldade em diferenciar colapso súbito de outras causas (ex.: fadiga extrema, convulsão, trauma). **A regra é tratar todo atleta caído e não responsivo como uma PCR até que se prove o contrário**;
- Ação: verificar responsividade (sacudir os ombros, chamar em voz alta). Se não responder, gritar por ajuda e acionar imediatamente o serviço de emergência médica (SAMU 192). Designar uma pessoa específica para fazer a ligação. Solicitar o DEA. Em locais esportivos, o DEA deve estar acessível e sinalizado, com tempo de acesso ideal de menos de 3 minutos.

### 7.2. Reanimação Cardiopulmonar Precoce de Alta Qualidade

- Adaptação esportiva: a RCP pode ser iniciada imediatamente por profissionais presentes (treinadores, preparadores físicos, colegas treinados, árbitros e inclusive atletas). A técnica é a mesma orientada pela AHA e SBC;
- Ação (básico):
  1. Abrir as vias aéreas (manobra fronte-mento);
  2. Verificar a respiração; se não estiver respirando ou se estiver ofegante (respiração agônica), iniciar compressões;
  3. Compressões torácicas: frequência de 100–120/min, profundidade de 5–6 cm, permitindo retorno completo do tórax. Minimizar interrupções;
  4. Ventilações: se treinado e disposto, realizar a razão 30:2 (compressões: ventilações); se não treinado ou relutante, fazer apenas compressões contínuas.

### 7.3. Desfibrilação Rápida

- Adaptação esportiva: a causa mais comum de PCR súbita no atleta é a FV. O choque é o único tratamento efetivo. O DEA deve chegar em até 3 minutos;
- Ação (básico/avançado):
  1. Ligar o DEA e seguir as instruções verbais;
  2. Rasgar a camisa, secar o tórax suado rapidamente, se necessário, para garantir a adesão dos eletrodos;
  3. Afastar todos da vítima durante a análise do ritmo e o choque; aplicar o choque, se indicado, e retomar imediatamente a RCP.

### 7.4. Suporte Avançado de Vida e Cuidados Pós-Parada

- Adaptação esportiva: realizado pela equipe do SAMU/emergência médica que estiver em campo, mas a equipe esportiva mandante deve facilitar o acesso da ambulância, e a transferência deve ser feita para hospital de referência previamente definido;
- Ação (avançado – por profissionais de saúde):
  1. Via aérea avançada: intubação endotraqueal ou dispositivo supraglótico, para garantir oxigenação e ventilação adequadas;
  2. Acesso venoso: obter acesso IO ou IV para a administração de medicamentos;
  3. Farmacologia: administrar adrenalina 1 mg IV/IO a cada 3–5 minutos durante a RCP. Se a FV/TV persistir, considerar amiodarona 300 mg IV/IO;
  4. Manejo de causas reversíveis – 5Hs (hipovolemia, hipóxia, hipotermia, hidrogênio (acidose), hipo-/hipercalcemia) e 5Ts (tóxicos, tamponamento cardíaco, tensão no tórax, trombose coronária, tromboembolismo pulmonar): no esporte, deve-se suspeitar fortemente de:
    – *Commotio Cordis*: trauma contuso no tórax (ex.: bola, impacto). O tratamento é a desfibrilação imediata;
    – Hipotermia/hipertermia: em esportes de *endurance*;
    – Distúrbios eletrolíticos: desidratação extrema.
  5. Pós-ressuscitação: após o RCE, iniciar suporte ventilatório, controle da temperatura alvo (32–36 °C – hipotermia terapêutica) e transporte rápido para um hospital com capacidade de intervenção coronária percutânea e cuidado integral.

### 7.5. Protocolo Específico para o Local de Treino/Competição

1. Prevenção: triagem cardiovascular (histórico e exame físico) para identificar atletas em risco;
2. Preparação: ter equipe treinada e um DEA disponível, testado e com bateria carregada. Ter um plano de ação escrito e conhecido por todos da equipe;
3. Simulação: realizar treinamentos e simulações regulares (a cada 6 meses) para toda a equipe, desde o segurança até o médico.

## Data Availability

os conteúdos subjacentes ao texto do Posicionamento estão contidos no manuscrito.

## Lista de abreviaturas e siglas

ACLS: Suporte avançado de vida em cardiologia (*advanced cardiac life support*)
AHA: American Heart Association
APH: Atendimento pré-hospitalar
APP: Avaliação pré-participação esportiva
CAV: Cardiomiopatia arritmogênica ventricular
CCM: Centro de comando médico
DAC: Doença arterial coronariana
DEA: Desfibrilador externo automático
EACPR: European Association of Cardiovascular Prevention and Rehabilitation
ECG: Eletrocardiograma
ERC: European Resuscitation Council
ESC: European Society of Cardiology
FV: Fibrilação ventricular
HEPA: Ar de partículas de alta eficiência (*high-efficiency particulate air*)
IO: Intraósseo
IV: Intravenoso
MCH: Miocardiopatia hipertrófica
MSC: Morte súbita cardíaca
PAE: Plano de ação de emergência
PAM: Plano de ação médica
PCR: Parada cardiorrespiratória
PMA: Posto médico avançado
RCE: Retorno da circulação espontânea
RCP: Reanimação cardiopulmonar
SADS: Síndrome da morte súbita arrítmica (*sudden arrhythmic death syndrome*)
SAMU: Serviço de Atendimento Móvel de Urgência
SAV: Suporte avançado de vida
SBC: Sociedade Brasileira de Cardiologia
SBME: Sociedade Brasileira de Medicina do Esporte
SBV: Suporte básico de vida
SCA: Parada cardíaca súbita (*sudden cardiac arrest*)
TECA: Treinamento de emergências cardiovasculares avançado
TV: Taquicardia ventricular
UTI: Unidade de terapia intensiva
