# Supplementary material for: Posicionamento sobre Emergências Cardiovasculares para Eventos Esportivos – 2026
Source: Arq Bras Cardiol. 2026 Apr 28;123(4):e20260220. [Article in Portuguese] doi: 10.36660/abc.20260220 (PMC13155795; doi:10.36660/abc.20260220)
Supplement: Translation

# Translation: Position Statement on Cardiovascular Emergencies at Sporting Events – 2026

**DOI:** 10.36660/abc.20260220i

**Authors:** Luiz Eduardo Fonteles Ritt, Antonio Carlos Avanza, Ricardo Stein, Rodrigo Otavio Bougleux Alô, Marcelo Bichels Leitão, Arthur Haddad Herdy, Marconi Gomes, Carolina Christianini Mizzaci, Anderson Donelli da Silveira, Fabio de Freitas Guimarães Guerra, Fabricio Braga, Milena dos Santos Barros Campos, Ricardo Galesso Cardoso, Nivaldo Filgueiras, Mateus Freitas Teixeira, Marcos Machado Barojas, Cléa Simone Sabino de Souza Colombo, Luciana Diniz Nagem Janot de Matos

**Affiliations:** 1 Salvador BA Brazil Escola Bahiana de Medicina e Saúde Pública, Salvador, BA – Brazil; 2 Salvador BA Brazil Instituto D’Or de Pesquisa e Ensino, Salvador, BA – Brazil; 3 Salvador BA Brazil Hospital Cárdio Pulmonar, Salvador, BA – Brazil; 4 ES Brazil Universidade Vila Velha, ES – Brazil; 5 RS Brazil Universidade Federal do Rio Grande do Sul, RS – Brazil; 6 São Paulo SP Brazil Instituto Dante Pazzanese de Cardiologia, São Paulo - SP – Brazil; 7 Curitiba PR Brazil IEMEX - Instituto de Endocrinologia e Medicina do Exercício, Curitiba, PR – Brazil; 8 Florianópolis SC Brazil Instituto de Cardiologia de Santa Catarina, Florianópolis, SC – Brazil; 9 Belo Horizonte MG Brazil Prefeitura de Belo Horizonte, Belo Horizonte, MG – Brazil; 10 Porto Alegre RS Brazil Hospital de Clinicas de Porto Alegre, Porto Alegre, RS – Brazil; 11 Porto Alegre RS Brazil Programa de Pós-Graduação em Cardiologia Universidade Federal do Rio Grande do Sul, Porto Alegre, RS – Brazil; 12 Porto Alegre RS Brazil Hospital Moinhos de Vento, Porto Alegre, RS – Brazil; 13 Santos SP Brazil Hospital Santa Casa de Santos, Santos, SP – Brazil; 14 Universidade Metropolitana de Santos, Santos, SP – Brazil; 15 Rio de Janeiro RJ Brazil Laboratório de Performance Humana, Rio de Janeiro, RJ – Brazil; 16 Aracajú SE Brazil "Hospital Universitário de Sergipe, Aracajú, SE – Brazil; 17 Aracajú SE Brazil Hospital São Lucas Rede D’Or São Luiz, Aracajú, SE – Brazil; 18 São Paulo SP Brazil Einstein Hospital Israelita, São Paulo, SP – Brazil; 19 Salvador BA Brazil Universidade do Estado da Bahia, Salvador, BA – Brazil; 20 Niterói RJ Brazil Clínica Fit Center e Club de Regatas Vasco da Gama, Niterói, RJ – Brazil; 21 Salvador BA Brazil Hospital Português, Salvador, BA – Brazil; 22 Campinas SP Brazil Faculdade de Medicina São Leopoldo Mandic, Campinas, SP – Brazil

**Table:** 

Position Statement on Cardiovascular Emergencies at Sporting Events – 2026	
The report below lists declarations of interest as reported to the SBC by the experts during the period of the development of these statement, 2025/2026.	
Expert	Type of relationship with industry
Anderson Donelli da Silveira	Nothing to be declared
Antonio Carlos Avanza Junior	Nothing to be declared
Arthur Haddad Herdy	Financial declaration B - Research funding under your direct/personal responsibility (directed to the department or institution) from the brazilian or international pharmaceutical, orthosis, prosthesis, equipment and implants industry: Janssen: Melvexian; Novartis: Inclisiran.
Carolina Christianini Mizzaci	Nothing to be declared
Cléa Simone Sabino de Souza Colombo	Nothing to be declared
Fabio de Freitas Guimarães Guerra	Financial declaration A - Economically relevant payments of any kind made to (i) you, (ii) your spouse/partner or any other person living with you, (iii) any legal person in which any of these is either a direct or indirect controlling owner, business partner, shareholder or participant; any payments received for lectures, lessons, training instruction, compensation, fees paid for participation in advisory boards, investigative boards or other committees, etc. from the brazilian or international pharmaceutical, orthosis, prosthesis, equipment and implants industry: AstraZeneca: Forxiga, Lokelma, Breztri. Other relationships Funding of continuing medical education activities, including travel, accommodation and registration in conferences and courses, from the brazilian or international pharmaceutical, orthosis, prosthesis, equipment and implants industry: Novo Nordisk: Rybelsus.
Fabricio Braga	Nothing to be declared
Luciana Janot	Nothing to be declared
Luiz Eduardo Fonteles Ritt	Financial declaration A - Economically relevant payments of any kind made to (i) you, (ii) your spouse/partner or any other person living with you, (iii) any legal person in which any of these is either a direct or indirect controlling owner, business partner, shareholder or participant; any payments received for lectures, lessons, training instruction, compensation, fees paid for participation in advisory boards, investigative boards or other committees, etc. from the brazilian or international pharmaceutical, orthosis, prosthesis, equipment and implants industry: - Pfizer: amyloidosis; AstraZeneca: amyloidosis; Clinical research on lipids, heart failure, dyslipidemia: AstraZeneca, Novartis, Novo Nordisk, Boehringer. Other relationships Funding of continuing medical education activities, including travel, accommodation and registration in conferences and courses, from the brazilian or international pharmaceutical, orthosis, prosthesis, equipment and implants industry: - AstraZeneca: amyloidosis; Pfizer: amyloidosis.
Marcelo Bichels Leitão	Nothing to be declared
Marconi Gomes	Nothing to be declared
Marcos Machado Barojas	Nothing to be declared
Mateus Freitas Teixeira	Nothing to be declared
Milena dos Santos Barros Campos	Nothing to be declared
Nivaldo Filgueiras	Financial declaration A - Economically relevant payments of any kind made to (i) you, (ii) your spouse/partner or any other person living with you, (iii) any legal person in which any of these is either a direct or indirect controlling owner, business partner, shareholder or participant; any payments received for lectures, lessons, training instruction, compensation, fees paid for participation in advisory boards, investigative boards or other committees, etc. from the brazilian or international pharmaceutical, orthosis, prosthesis, equipment and implants industry: - AstraZeneca: Lokelma, Forxiga; Servier: Adhesion/Acertanlo, Acertil; Boehringer: Jardiance. Other relationships Funding of continuing medical education activities, including travel, accommodation and registration in conferences and courses, from the brazilian or international pharmaceutical, orthosis, prosthesis, equipment and implants industry: - Servier: Acertanlo; AstraZeneca: Lokelma
Ricardo Galesso Cardoso	Nothing to be declared
Ricardo Stein	Financial declaration A - Economically relevant payments of any kind made to (i) you, (ii) your spouse/partner or any other person living with you, (iii) any legal person in which any of these is either a direct or indirect controlling owner, business partner, shareholder or participant; any payments received for lectures, lessons, training instruction, compensation, fees paid for participation in advisory boards, investigative boards or other committees, etc. from the brazilian or international pharmaceutical, orthosis, prosthesis, equipment and implants industry: Life Genomics: TEB. B - Research funding under your direct/personal responsibility (directed to the department or institution) from the brazilian or international pharmaceutical, orthosis, prosthesis, equipment and implants industry: CNPq: FIPE/HCPA.
Rodrigo Otavio Bougleux Alô	Financial declaration A - Economically relevant payments of any kind made to (i) you, (ii) your spouse/partner or any other person living with you, (iii) any legal person in which any of these is either a direct or indirect controlling owner, business partner, shareholder or participant; any payments received for lectures, lessons, training instruction, compensation, fees paid for participation in advisory boards, investigative boards or other committees, etc. from the brazilian or international pharmaceutical, orthosis, prosthesis, equipment and implants industry: Libbs: pitavastatin. Other relationships Funding of continuing medical education activities, including travel, accommodation and registration in conferences and courses, from the brazilian or international pharmaceutical, orthosis, prosthesis, equipment and implants industry: Sanofi: congress.

## Table of contents

**1. Introduction: Sudden Death in Sports – Scope of the Problem** 5
  **1.1. Etiologies** 5
  **1.2. Epidemiology and Incidence** 7
  **1.3. Current Situation: Gaps Between Knowledge and Practice** 7
  **1.4. Rationale and Call to Action** 8
**2. Preparticipation Evaluation in Sports Cardiology** 8
  **2.1. Brazilian Context** 8
  **2.2. Recommendations of the Brazilian Society of Cardiology for Preparticipation Evaluation** 8
  **2.3. Recommended evaluation profile and standard** 9
**3. Checklist and Essential Equipment for Cardiovascular Emergencies at Major Sporting Events** 9
  **3.1. Structure of the Equipment Plan** 9
  **3.2. Basic life Support Equipment** 9
  **3.3. Advanced Life Support Equipment** 10
  **3.4. Communication and Operational Logistics** 10
  **3.5. Sport-Specific Equipment** 10
  **3.6. Inspection, Maintenance, and Training Procedures** 10
  **3.7. Final Considerations** 11
**4. Team Composition, Training, and Competency Requirements** 11
**5. Equipment, Location, and Logistics at Sporting Events** 11
  **5.1. Introduction** 11
  **5.2. Essential Equipment** 13
    **5.2.1. Automated External Defibrillators** 13
    **5.2.2. Advanced Life Support Equipment** 13
    **5.2.3. Complementary Resources** 13
  **5.3. Care Location and Infrastructure** 13
    **5.3.1. Internal Mapping** 13
    **5.3.2. Medical Rooms and Care Stations** 13
    **5.3.3. Internal and External Transport** 13
  **5.4. Operational Logistics** 13
    **5.4.1. Emergency Action Plan** 13
    **5.4.2. Team and Training** 13
    **5.4.3 Communication and Central Command** 14
  **5.5. Impact of Equipment and Logistics on Survival** 14
  **5.6. Operational Considerations for the Brazilian context** 14
  **5.7. Conclusion** 14
**6. Care Delivery, Evacuation, and Transport Logistics** 14
  **6.1. Planning and Medical Infrastructure** 15
    **6.1.1. Pre-Event Risk Assessment** 15
    **6.1.2. Care Structure** 16
    **6.1.3. Communication and Command** 16
    **6.1.4. Event Operations** 17
    **6.1.5. Advanced Medical Posts** 17
    **6.1.6. Mobile Teams** 17
    **6.1.7. Communication and Command Cener** 17
  **6.2. Medical Care and Triage** 17
    **6.2.1. Logistics During Medical Care** 17
    **6.2.2. Documentation and Quality Indicators** 17
  **6.3. Medical Evacuation** 17
    **6.3.1. Evacuation Criteria** 17
    **6.3.2. Evacuation Routes** 17
    **6.3.3. Integration with Referral Hospitals** 18
    **6.3.4. Safe Transport** 18
    **6.3.5. Monitoring During Transport** 18
  **6.4. Legal and Safety Aspects** 18
  **6.5. Considerations by Environment** 18
**7. Basic and Advanced Life Support Protocols (the Chain of Survival) Adapted to Sports Settings** 18
  **7.1. Immediate Recognition and Activation of Emergency Medical Services** 18
  **7.2. Early, High-Quality Cardiopulmonary Resuscitation** 19
  **7.3. Rapid Defibrillation** 19
  **7.4. Advanced Life Support and Post-Cardiac Arrest Care** 20
  **7.5. Site-Specific Protocol for Training and Competition Venues** 20
**References** 20

## 1. Introduction: Sudden Death in Sports – Scope of the Problem

Sudden death is traditionally defined as an unexpected fatal event, whether witnessed or not, occurring within 1 hour of symptom onset or 24 hours of the individual being last seen in good health and without clinical complaints.^1^ In the sports setting, sudden death is considered related to physical activity if the event occurs within 1 hour of moderate- to high-intensity exercise.^2^ In athletes, often viewed as icons of health, such events carry profound emotional and social repercussions, affecting families, teams, spectators, and sports institutions. The complexity of this issue stems from the multiple variables involved, including sex, age, ethnicity, body characteristics, sport modality, playing position, training intensity, and environmental factors.

### 1.1. Etiologies

The causes of sudden death in athletes may be congenital or acquired. In younger individuals (< 35 years of age), genetic structural heart diseases and channelopathies predominate, along with viral myocarditis.^3-6^ In athletes aged 35 years or older, coronary artery disease (CAD) accounts for more than 80% of cases of sudden cardiac death (SCD).^7-9^

In some cases, autopsy reveals a structurally normal heart, characterizing the so-called sudden arrhythmic death syndrome (SADS).^3-5^ Recent data from registries in the United States, United Kingdom, and Australia suggest that SADS has been reported as a frequent cause of sudden death in young athletes.^6^ Nevertheless, structural diseases such as hypertrophic cardiomyopathy (HCM) and arrhythmogenic ventricular cardiomyopathy (AVC) remain among the leading etiologies associated with sudden death in this population. Acquired causes include viral myocarditis, which deserves particular attention, as it has become increasingly prevalent among young athletes and is often related to common viruses such as influenza, coxsackievirus, and parvovirus.

Because myocardial inflammation is frequently underestimated clinically and athletes often continue training despite infectious symptoms, it may go unnoticed and result in permanent sequelae. Following the emergence of COVID-19, the incidence of SARS-CoV-2-induced myocarditis has raised additional concern, particularly among individuals engaged in high-intensity physical activity.

It is important to emphasize that not all sudden deaths in athletes are of cardiac origin. The widespread use of performance-enhancing substances – such as anabolic steroids, stimulants (amphetamines, energy drinks), peptide hormones, and illicit recreational drugs (eg, cocaine) – has been associated with an increased risk of acquired cardiovascular disease and sudden death in young and middle-aged individuals.^10^ Other non-cardiac causes also warrant attention, including hyperthermia, fluid and electrolyte disturbances, chest trauma, concussion, drowning, and sport-specific syncope.^11^

**Figure:** AED: automated external defibrillator; ALS: advanced life support; CPR: cardiopulmonary resuscitation; EAP: emergency action plan; ECG: electrocardiogram; EMS: Emergency Medical Services.

### 1.2. Epidemiology and Incidence

The true incidence of sudden death in athletes remains uncertain, partly due to the heterogeneity of available data sources, which often rely on media reports and insurance records, as well as the diversity of populations studied. It is estimated that 56% to 80% of sudden deaths in young athletes occur during sports participation, with reported incidences ranging from 1:1,000,000 to 1:5,000 athlete-years.^3,12,13^

Certain groups are at increased risk, including male athletes, individuals of African descent, and basketball or soccer players. Men have a higher relative risk than women (3:1 to 9:1), while athletes of African descent have an approximately 3.2-fold higher risk compared with White athletes.

Among basketball players, the annual incidence of sudden death reaches 1:9,000 in White men and 1:5,300 in men of African descent.^3,12,14,15^

Preparticipation physical evaluation (PPE) remains the cornerstone of prevention of sudden death in sports, allowing for the identification of at-risk individuals and the implementation of management strategies.

In addition, the establishment of emergency protocols and rapid response to cardiovascular collapse are determinants of favorable outcomes.

### 1.3. Current Situation: Gaps between Knowledge and Practice

Despite substantial advances in the understanding of the pathophysiology of sudden death in the sporting context and the availability of well-established diagnostic and therapeutic tools, a critical gap persists between theory and practice in many settings. Studies show that cardiac arrests occurring during or immediately after sporting events are still frequently managed inadequately, resulting in unfavorable outcomes that could often be prevented.^16,17^

In Brazil, this reality has particular features. While some developed countries have established robust structures for cardiovascular emergency response at sporting events, a large proportion of events held nationwide lack standardized protocols, adequately trained personnel to promptly recognize and respond to cardiac arrest, strategically positioned automated external defibrillators (AEDs), and widespread cardiopulmonary resuscitation (CPR) training among organizers and security staff. Concurrently, medical PPE, an essential tool for identifying individuals at risk and preventing sudden death at its origin, remains inconsistently implemented across different contexts and sports modalities.^12^

The urgency of this intervention is further underscored by critical pathophysiological considerations. For each minute of delay in initiating CPR, survival decreases by approximately 6% to 10%, reflecting a progressive reduction in the likelihood of return of spontaneous circulation (ROSC).^16,18^ Even more importantly, the optimal window for intervention occurs in the first few minutes after collapse, when shockable rhythms – ventricular tachycardia (VT) and ventricular fibrillation (VF) – are more frequent and have the highest success rates with defibrillation.^19^ Beyond this critical window, there is a progressive shift toward asystole or pulseless electrical activity, both non-shockable rhythms associated with a substantially more guarded prognosis and lower survival rates.^18,19^

Guidelines such as those issued by the Brazilian Society of Sports Medicine (SBME) in 2005 established robust and well-founded recommendations for the prevention and management of sudden death in sports.^19^ However, nearly 2 decades later, a substantial gap remains between these recommendations and their practical implementation at Brazilian sporting events.

A systematic assessment of adherence to the 2005 SBME recommendations across different Brazilian sporting contexts reveals critical deficiencies in multiple dimensions (Table 1).^19^

**Table 1:** Recommendations for reducing sudden death at sporting events: implementation and clinical impact

Dimension	Level of implementation	Clinical impact
Standardized PPE	30%–40%	CRITICAL – Failure to identify risk
*On-site defibrillators*	50%–60%	CRITICAL – Missed window of opportunity
CPR training	25%–35%	CRITICAL – Delay in response
Protocols	40%–50%	HIGH – Heterogeneity of approaches
Integration with EMS	20%–30%	HIGH – Delay in advanced features
Centralized registration	10%–15%	HIGH – Epidemiological inaccuracy
Legislation implemented	35%–45%	MEDIUM – Lack of oversight

CPR: cardiopulmonary resuscitation; EMS: Emergency Medical Services; PPE: preparticipation evaluation.

The most critical shortcomings are concentrated in 3 fundamental pillars:

- Primary prevention through appropriate PPE;
- Rapid response infrastructure with defibrillators and trained teams;
- Standardized protocols integrated with emergency medical systems.

This fragmented implementation results in marked variability in care across events and institutions, undermining equity in athlete protection.

### 1.4. Rationale and Call to Action

Within this context, initiatives such as this Position Statement on cardiovascular emergencies at major sporting events, led by the Department of Exercise Testing, Rehabilitation, Sports Cardiology, and Nuclear Cardiology of the Brazilian Society of Cardiology (SBC), assume strategic relevance by proposing a reference framework that integrates both cardiovascular prevention and emergency response.

The convergence of efforts among specialist medical societies, combined with the decisive involvement of event organizers, sports institutions, and public authorities, is imperative to translate knowledge into institutionalized action and save lives.

This document represents a necessary advance in the development of public policies and operational guidelines that enable not only early detection of individual risk but also the adequate preparedness of the entire sporting event ecosystem to respond effectively to cardiovascular emergencies, thus reducing preventable mortality and ensuring that sports participation is safe for all participants.

## 2. Preparticipation Evaluation in Sports Cardiology

The primary goal of cardiovascular PPE is to identify potentially life-threatening cardiovascular conditions that may increase the risk of SCD and malignant arrhythmias during sports participation. PPE is recommended for competitive athletes, recreational sports participants, and individuals initiating high-intensity training.

This assessment enables early detection of structural heart disease, channelopathies, and other high-risk conditions and is therefore essential for ensuring safety in sports practice.^12,21,22^

### 2.1. Brazilian Context

In Brazil, PPE is more common in sports clubs (such as soccer clubs) and in endurance competitions (such as running races, trail running, and triathlon), which require mandatory medical clearance. The Brazilian Federal Board of Medicine establishes that the evaluation should include:

- Detailed medical history;
- Comprehensive physical examination;
- Complementary testing (when indicated).

Both SBC and SBME recommend protocols adapted to the national context, considering cost-effectiveness, disease prevalence, and available infrastructure.^21^

The Brazilian model incorporates elements of the North American protocol (such as the 14-point history and physical examination – Table 2) and the European protocol, which includes resting electrocardiogram (ECG) as a mandatory test.

**Table 2:** The 14-point preparticipation evaluation

Category	Point	Criterion
Medical history (8 points)	1	Chest pain/discomfort during exercise
	2	Syncope or near-syncope related to exertion
	3	Dyspnea or excessive disproportionate fatigue
	4	History of heart murmur
	5	Diagnosed arterial hypertension
	6	Family history of sudden death (< 50 years of age)
	7	Family history of cardiomyopathy, Marfan syndrome, or severe arrhythmias
	8	Congenital heart disease in first-degree relatives
Physical examination (6 points)	9	Pathological heart murmurs
	10	Diminished femoral pulses (suspected aortic coarctation)
	11	Signs of Marfan syndrome (increased arm span, chest wall deformities)
	12	Blood pressure measurement
	13	Abnormal heart sounds (S3, S4, or gallop rhythm)
	14	Arrhythmias detected on examination

PPE is, therefore, a key preventive strategy and should be conducted by physicians with appropriate expertise, using a risk-benefit-based approach aligned with national and international guidelines.

An important aspect of PPE, to avoid unnecessary additional testing, is the recognition of ECG and imaging abnormalities that reflect training adaptations inherent to the athlete. International criteria for ECG interpretation in athletes have been published and should be followed during evaluations.^23,24^

### 2.2. Recommendations of the Brazilian Society of Cardiology for preparticipation evaluation

1. Initial evaluation
  - Detailed medical history (personal and family history);
  - Comprehensive physical examination (with attention to risk factors and clinical signs);
  - ECG (essential for identifying suspicious electrical abnormalities).^23^
2. Complementary testing (based on clinical judgment)
  - Exercise stress testing (assessment of exercise response, particularly in adults at moderate or high risk);
  - Echocardiography (identification of cardiomyopathies and structural abnormalities);
  - Laboratory tests (complete blood count, glucose, lipid profile, and renal function, among others);
  - Cardiac magnetic resonance imaging (in selected cases).^24^

### 2.3. Recommended evaluation profile and standard

- Age < 35 years, without comorbidities: medical consultation + ECG;
- Age ≥ 35 years or with cardiovascular risk factors: medical consultation + ECG + laboratory tests + exercise stress testing or other functional/anatomical testing according to the pretest probability of CAD;
- Elite athletes: comprehensive evaluation (ECG, echocardiography, functional testing – exercise stress testing or cardiopulmonary exercise testing).

## 3. Checklist and Essential Equipment for Cardiovascular Emergencies at Major Sporting Events

The management of cardiovascular emergencies at major sporting events requires integrated planning, multiprofessional training, and adequate infrastructure to ensure a rapid and effective response to cardiac arrest or other acute cardiac emergencies.^25-27^

A well-structured emergency action plan (EAP) must ensure rapid response, integration among medical teams, and the availability of equipment that enables immediate and effective care. This plan should consist of written protocols that are periodically tested and represents the central pillar of any prehospital care strategy in these settings.^28^

Cardiovascular emergencies occurring on the field may affect not only apparently healthy athletes – due to conditions such as cardiomyopathies, coronary artery anomalies, long QT syndrome, or arrhythmogenic right or left ventricular cardiomyopathy – but also members of the technical staff and spectators.^26,29^

Current evidence recommends that the interval between collapse and the first defibrillation shock should not exceed 3 minutes.^29^ Therefore, equipment placement and team training directly determine post-cardiac arrest survival rates, particularly in high-density environments, such as stadiums, arenas, and athletic tracks.^28^

### 3.1. Structure of the Equipment Plan

The equipment arsenal must cover the full spectrum of life support:

- Basic life support (BLS) – directed toward first responders and ancillary staff;
- Advanced life support (ALS) – delivered by qualified health care professionals;
- Logistics and trauma support – intended for immobilization, transport, and management of associated injuries.

Equipment should be organized into modular kits (airway, ventilation, circulation, medications, and trauma) within clearly identified bags or backpacks, with mandatory pre-event checklists.^29^ The use of printed or digital protocols is also recommended to standardize the management of cardiac arrest, ROSC, and collapsed athlete.

The materials must encompass resources ranging from BLS to ALS in cardiology, as well as communication devices, transport resources, and hospital logistics.^27,29^ The equipment list should be adapted according to the sport modality, event size, number of participants, and expected response time.

All equipment must be inspected before each event, tested for functionality, and documented using digital or printed checklists.^30^

### 3.2. Basic Life Support Equipment (Table 3)

**Table 3:** Basic life support equipment

AED	Essential device for early defibrillation. Must be visibly and easily accessible within 3 minutes from any point in the venue.^29^ Batteries charged, spare pads available, and pre-event checks are recommended.
Bag-valve-mask (Ambu bag)	Essential for manual ventilation in cases of apnea. Must be available at all care points.
Face masks and HEPA filters	Used to prevent cross-contamination and ensure safe ventilation.
Oropharyngeal and nasopharyngeal airways (various sizes)	Maintain airway patency during CPR and facilitate effective ventilation.
Scissors, razor blade, disposable towels	Allow rapid chest exposure and AED pad placement.
Portable pulse oximeter	Assists in initial monitoring of oxygen saturation and heart rate during evaluation.

AED, automated external defibrillator; CPR, cardiopulmonary resuscitation; HEPA, high-efficiency particulate air.

### 3.3. Advanced Life Support Equipment (Table 4)

**Table 4:** Advanced life support equipment

Equipment	Description and application
Manual monitor-defibrillator and transcutaneous pacemaker	Enables rhythm analysis, waveform recording, and manual defibrillation by trained personnel.^26^ Transcutaneous pacemaker with adhesive pads is essential for atrioventricular block management.
Portable oxygen source (≥ 5 L/min)	Includes non-rebreather masks, nasal cannulas, Venturi devices for proper supplementation (with their appropriate connectors), and portable oxygen cylinders.
Laryngeal masks and endotracheal tubes (6.0–8.5 mm)	Alternatives for advanced airway management when bag-valve-mask ventilation fails.
Portable suction device	Essential to prevent aspiration during CPR maneuvers.
Peripheral and intraosseous vascular access	Includes catheters, infusion sets, and needles for rapid administration.
Emergency medications	Must include epinephrine, amiodarone, atropine, dopamine, lidocaine (in ampoules), nitroglycerin, aspirin, 50% magnesium sulfate (in ampoules), sodium bicarbonate (in ampoules), hypertonic glucose, and 0.9% saline solutions.^25,27^
Automated chest compression device	Optional but useful during prolonged CPR, especially in large venues.^28,29^

CPR: cardiopulmonary resuscitation.

### 3.4. Communication and Operational Logistics

- Dedicated radio communication and closed communication systems between medical and security teams;
- Standardized emergency code protocols with clearly defined roles;
- Visual maps indicating care points, ambulance routes, and AED locations;
- Ambulance equipped as a mobile intensive care unit (ICU) with an ALS-trained cardiac team (advanced cardiac life support [ACLS]) and a designated area for rapid field access;^30,31^
- Clear visual identification of medical staff (vests, badges) and direct communication with the referral hospital for post-event coordination.

### 3.5. Sport-Specific Equipment (Table 5)

**Table 5:** Sport-specific equipment

Sport	Specific equipment	Clinical rationale
Soccer	AED, rigid boards, cervical collars, portable suction, advanced airway kit.	High incidence of collapses due to malignant arrhythmias and chest trauma.
Basketball	AED courtside, oxygen kit, splints, immobilizers.	Increased risk of cardiomyopathy-related cardiac arrest and blunt chest trauma.
Athletics	Mobile AED and oxygen stations every 200–300 m.	Predominance of vasovagal syncope, heat exhaustion, and exertional VF.
Swimming	AED and airway devices near pool, flotation board, waterproof cervical collar, materials to dry the chest before pad placement.	Rapid water removal, chest drying for AED use, and immediate ventilation are critical.^26,29^
Martial arts	AED near tatami mats, intubation kit, cervical collar, facial protection, portable suction.	Risk of cervical trauma, concussion, and post-impact reflex apnea.
Cycling/triathlon	On-site ambulance, support vehicles (motorcycles, bicycles, boats, kayaks) with portable AED and radio communication system.	Long courses require distributed response points and logistical mobility.^29,31^

AED: automated external defibrillator; VF: ventricular fibrillation.

### 3.6. Inspection, maintenance, and training procedures

Pre-event equipment checks, supervised by the event's medical coordinator, should follow a documented protocol, including:

- Battery charge verification, defibrillator testing, medication expiration checks, cable integrity, and oxygen cylinder inspection;
- Quarterly operational simulations involving all teams (physicians, physiotherapists, referees, security staff);
- Digital or printed checklists filed by the event's medical coordinator;^30^
- Defined hospital routes and referral centers with ACLS.

Periodic CPR and AED training for all involved professionals and staff is mandatory, in accordance with the American Heart Association (AHA) and European Resuscitation Council (ERC) recommendations.^25,26,29^

Local health authorities (eg, health surveillance agencies) should conduct on-site audits to ensure compliance and adequacy.

### 3.7. Final Considerations

Effective response to cardiovascular emergencies at major sporting events depends on the integration of 3 fundamental pillars:

1. Strategic care planning (with a tested and updated EAP);
2. Adequate and functional equipment;
3. Regular multiprofessional team training.

The implementation of sport-specific checklists and monitoring of logistical readiness significantly reduce time to defibrillation and improve survival outcomes. Medical teams must be prepared not only for ALS but also for trauma, hyperthermia, and anaphylaxis management, conditions frequently encountered in these settings. Regular equipment inspection and interdisciplinary training are non-negotiable pillars to ensure the safety of athletes and spectators.

Preventing avoidable sudden deaths among athletes and the public therefore requires a systematized response model, grounded in evidence-based protocols and continuous quality improvement.^27,30,32^

## 4. Team Composition, Training, and Competency Requirements

At major sporting events, a medical director or coordinator is required and is responsible for developing an action plan for the management of cardiovascular emergencies, establishing up-to-date protocols and serving as the interface between first responders and Emergency Medical Services (EMS) to which the patient may ultimately be transferred.

SBC offers life support training programs at multiple levels, including courses for laypersons (advanced cardiovascular emergency training for laypersons – TECA L) and for health care professionals at the basic (TECA B) and advanced (TECA A) levels.^33-36^

The medical director must hold an active license with the Regional Board of Medicine, have experience in emergency care and in the management of medical services at events, and be formally trained in advanced cardiovascular emergency care (TECA A).^35^

The number of health care professionals involved in medical coverage should be proportional to the total number of participating athletes and must comply with applicable local regulations. The on-site medical team (whether in ambulances or fixed medical aid stations) should preferably include a physician, a nurse, and a nursing technician, in addition to the ambulance driver.^33-36^

The physician must have an active license with the Regional Board of Medicine and, ideally, training and/or experience in one of the following areas: emergency medicine, intensive care, sports medicine, orthopedics, or cardiology. Advanced training in cardiovascular emergency care (eg, TECA A) is mandatory.^35^

The nurse should likewise have training and experience in emergency care and be certified in advanced cardiovascular emergency care (eg, TECA A). The nursing technician should have emergency care experience and be trained in basic cardiovascular emergency care (TECA B). Ambulance drivers should ideally receive training in cardiovascular emergencies (TECA L). Depending on the logistics, type of sporting event, and EAP, first responders or civilian firefighters trained in first aid and BLS may also be incorporated into the rapid response team.^33-36^

For events involving professional clubs or teams with their own medical staff to care for athletes, the team physician must hold an active license with the Regional Board of Medicine, have training in emergency medicine, cardiology, orthopedics, or sports medicine, and be certified in basic cardiovascular emergency care (TECA B). In addition, the EAP, the composition of the response team, and the location of defibrillators (automated or manual) must be defined in advance. Ideally, the specific roles and responsibilities of each team member in case of an emergency should be clearly established prior to the event.

It is strongly recommended that technical staff members, and even athletes themselves, receive BLS training (at a minimum TECA L, preferably TECA B), as they may often be the first to witness a collapse. They should be able to recognize a potential cardiac arrest, initiate CPR maneuvers, and promptly activate emergency assistance and retrieve an AED.

## 5. Equipment, Location, and Logistics at Sporting Events

### 5.1. Introduction

Major sporting events, whether professional or recreational, bring together large numbers of athletes and spectators in environments characterized by high emotional, physiological, and logistical demands. This combination creates a setting conducive to an increased risk of cardiovascular emergencies, particularly cardiac arrest.

Recent studies demonstrate that, although the incidence of cardiac arrest in sports arenas is relatively low, survival depends almost exclusively on the prompt availability of AEDs, trained personnel, and well-organized logistics for immediate care. The interval between collapse and defibrillation is a key determinant of survival and should ideally be less than 3 minutes. Each 5-minute delay in ROSC in patients with out-of-hospital cardiac arrest is associated with a 38% increase in mortality risk.^37-39^

Therefore, the position statement of SBC on cardiovascular emergencies at major sporting events emphasizes 3 foundational pillars: equipment availability, training and competency, and strategic placement with integrated logistics. These principles are grounded in national and international evidence and in the experience of cardiovascular safety programs implemented in European and North American arenas (Figure 1).

**Figure 1:** List of equipment and medications required for medical emergencies at major sporting events.

### 5.2. Essential Equipment

#### 5.2.1. Automated External Defibrillators

Each arena or event venue should have a detailed map identifying evacuation routes, the location of AEDs, medical rooms, and ambulance access points. This document must be incorporated into the event's operational plan and serve as a reference for all emergency response teams.

Strategic AED distribution is the most effective measure to reduce mortality from cardiac arrest at sporting events. The European Society of Cardiology (ESC) recommends a maximum interval of 3 minutes between collapse and delivery of the first shock, which requires an adequate number of devices positioned equidistantly throughout the stadium or along the course, calculated according to area and average travel time. European guidelines suggest: 1–2 AEDs for arenas hosting up to 10,000 spectators; 4 AEDs for those with 10,000–50,000 spectators; and 8 or more AEDs for arenas exceeding 50,000 spectators.^40^

AEDs must be clearly signposted, routinely checked for functionality (including battery and adhesive pads), and placed near emergency access points and areas of high crowd density, such as central stands, VIP areas, circulation corridors, and press zones.

#### 5.2.2. Advanced Life Support Equipment

In addition to AEDs, the infrastructure should include (see Section 3):

- Supplemental oxygen and bag-valve-mask (Ambu bag);
- Cardiac monitors and pulse oximeters;
- Airway management kits (oropharyngeal airways, laryngoscope, endotracheal tubes);
- ALS medications (epinephrine, amiodarone, lidocaine, and saline solutions, among others);
- Stretchers, spine boards, and transport chairs;
- Cooling kits for the management of hyperthermia.

#### 5.2.3. Complementary Resources

Communication equipment (radios, centralized coordination systems), ambulance access, and fully equipped medical rooms are essential components of the minimum structure required to ensure safety in large arenas.

### 5.3. Care Location and Infrastructure

#### 5.3.1. Internal Mapping

Each arena or competition venue should have a detailed map showing evacuation routes, AED locations, medical aid stations, and ambulance access points. This document should be part of the medical action plan (MAP), which must be reviewed and widely disseminated prior to each event. Critical points include:

- Main and service gates with reserved space for ambulances;
- Areas of difficult access (upper stands, organized supporter sections);
- Athlete and officiating areas with direct access to medical units.

#### 5.3.2. Medical Rooms and Care Stations

At least one medical room per 10,000 spectators should be available, equipped for ALS and with direct ambulance access. In arenas or events hosting more than 50,000 spectators, additional sector-based medical rooms are recommended. These units must be clearly visible, adequately signposted, and integrated into communication systems and local EMS protocols.

#### 5.3.3. Internal and External Transport

Patient transfer to the referral hospital should occur within 5 minutes. Inside the arena, unobstructed access routes must be ensured, as well as the availability of electric vehicles or motorized stretchers for transport in hard-to-reach areas. Outside the arena, at least one ALS ambulance (mobile ICU) per 10,000 spectators is recommended, integrated with direct coordination with the referral hospital.

Event organizers must ensure an on-site prehospital care service, with ambulances positioned at the venue and capable of providing initial stabilization and immediate transport to the referral center. This system may operate independently of public prehospital care services (such as EMS or Fire Department), although integration and support from these institutions should be available in complex situations, including mass casualty incidents.

### 5.4. Operational Logistics

#### 5.4.1 Emergency Action Plan

The MAP should include:

- The name and role of the event's medical director, responsible for all clinical decisions;
- A description of the medical team (physicians, nurses, nursing technicians, first responders, and volunteers);
- Activation and communication flowcharts;
- Locations of equipment and medical rooms;
- Care and triage protocols;
- Communication pathways with external services (EMS, Fire Department, referral hospitals);
- Documentation procedures and adverse event reporting.

The plan should be tested and simulated annually, including hands-on training with all teams involved.

#### 5.4.2 Team and Training

The European Association of Cardiovascular Prevention and Rehabilitation (EACPR), Sports Cardiology Section, recommends the presence of at least:

- 2 physicians per 50,000 spectators;
- 1 nurse per 10,000 spectators;
- 2 emergency technicians per 10,000 spectators;
- At least 50% of volunteers trained in CPR and AED use.^40^

In addition to the recommendations outlined in this document, all applicable local legislation must be observed.

All team members should wear visible identification, carry communication radios, and use standardized uniforms. Training should follow ERC protocols and include realistic simulations of cardiac arrest, with performance assessment and a debriefing meeting after each simulation or real event.^40^

#### 5.4.3 Communication and Central Command

The operations center should function as the core of medical coordination, with an exclusive radio channel, a direct line to the referral hospital, and real-time monitoring of all incidents. Pre-event testing of communication coverage and system redundancy is mandatory to ensure operational efficiency and optimal response times during the event.

##### 5.5. Impact of Equipment and Logistics on Survival

Robust evidence demonstrates that the presence of trained teams and AEDs dramatically reduces mortality:

- In arenas with AED availability and response times ≤ 3 minutes, survival rates reach 60%–70%, with complete neurological recovery in most cases. In venues without AEDs or structured plans, survival rarely exceeds 5%;^26,40,41^
- In a French study by Marijon et al., survival from cardiac arrest in sports facilities was 22.8%, compared with 8% outside such facilities, a difference attributed to the presence of witnesses, early resuscitation, and superior logistics;^42^
- Documented cases, such as those from the AZ Alkmaar soccer stadium (Netherlands), reported 100% survival in 4 consecutive cardiac arrests, all reversed with AED use and rapid transport;^41^
- Although the risk of cardiac arrest during marathons in the United States has remained stable over time, mortality has been reduced by nearly half since 2010. This advance reflects improvements in immediate emergency response, particularly early bystander CPR and rapid AED use, measures proven to increase survival in long-distance running races.^43^

##### 5.6. Operational considerations for the Brazilian context

At major national events, such as soccer matches, running races, triathlons, marathons, and hybrid events, the implementation of international guidelines should include:

- Integration of local EMS and Fire Department into pre-event protocols;
- Designation of a referral hospital with 24-hour cardiology capability;
- Coordination with sports authorities and law enforcement for crowd flow and safety control;
- Contingency planning for extreme weather conditions, with specific plans for intense heat and dehydration;
- Annual training of field physicians, physiologists, and athletic trainers for early recognition of signs of cardiovascular collapse.

##### 5.7. Conclusion

The organization of cardiovascular emergency care at major sporting events requires far more than the mere availability of a defibrillator: it demands a well-defined system, trained personnel, and standardized protocols. Appropriate equipment, strategic placement, and efficient logistics form an integrated chain of survival in which every minute is critical. Models proposed by the EACPR and AHA demonstrate that preparedness saves lives and that each arena or competition venue should be planned as a hospital micro-environment, with its own infrastructure and seamless communication with emergency systems.

Figure 2 illustrates the ideal placement of AEDs in a large stadium, ensuring complete coverage within a 3-minute response radius from any point in the stands or technical areas. Equidistant placement of devices ensures response times compatible with the chain of survival and optimizes the likelihood of successful resuscitation.

**Figure 2:** Optimal placement of automated external defibrillators (AEDs) in a large stadium

Figure 3 and Table 6 present the EACPR recommendations regarding equipment and personnel.

**Figure 3:** Proposed organizational chart for the medical team at sporting events. EMS: Emergency Medical Services.

**Tabela 6:** Recomendações de equipamentos e pessoal

Capacidade da arena	DEAs	Médicos	Enfermeiros	Técnicos	Ambulâncias
< 10.000	1–2	1	1	2	0–1
10–50.000	4	2	1–5	2–10	1–2
> 50.000	8+	2–4	> 5	> 10	> 2

DEAs: desfibriladores externos automáticos.

Figure 4 depicts the standardized sequence of medical response to cardiovascular collapse in sports arenas, integrating immediate recognition, CPR with AED use, arrival of the medical team, internal transport, transfer to a mobile ICU, and final communication with the referral hospital, followed by case documentation.

**Figure 4:** Flowchart of the medical action plan for cardiovascular emergencies. AED: automated external defibrillator; CPR: cardiopulmonary resuscitation; ICU: intensive care unit.

## 6. Care Delivery, Evacuation, and Transport Logistics

The organization of major sporting events poses challenges related to health care planning, public safety, and emergency medical response. Classified within the field of mass gathering medicine, this interdisciplinary area focuses on the health implications of events that bring together large numbers of people within a limited space and time, potentially generating health care demands that exceed local system capacity.^44^

In the sporting context, events such as marathons, triathlons, Olympic Games, world championships, and major football tournaments present heightened logistical complexity. These events involve not only athletes of varying ages and fitness levels but also spectators, volunteers, and support personnel. Cardiovascular emergencies represent a smaller proportion of medical incidents, yet they require immediate and highly coordinated responses.^45^ Such responses must be supported by integrated systems based on advanced planning, appropriate infrastructure, multidisciplinary teams, clear triage protocols, efficient communication, and hospital integration, aiming to minimize the interval between clinical collapse and definitive intervention, in accordance with the time-dependent principle of emergency care.

Sports physicians, cardiologists, orthopedists, and emergency/critical care physicians play a central role throughout this process, from pre-event planning and medical infrastructure design to immediate care delivery, stabilization of critically ill patients, and decision-making regarding evacuation and transport.

### 6.1. Planning and Medical Infrastructure

#### 6.1.1. Pre-Event Risk Assessment

Care planning should begin months before the competition and be based on a comprehensive risk assessment, including:

- Epidemiological profile of athletes (age range, prevalence of comorbidities, competitive level);
- Environmental conditions (climate, humidity, altitude, air pollution);
- History of incidents in similar previous events;
- Capacity of local health care services (hospitals, EMS, Fire Department).

A qualitative study conducted at the Athens Marathon demonstrated that interorganizational knowledge transfer among health care, security, and event management stakeholders was critical for aligning triage protocols, evacuation strategies, and hospital integration.^46^ This interinstitutional coordination is currently regarded as a key success factor in mass gathering medicine.

#### 6.1.2 Care Structure

Medical care infrastructure should include a combination of fixed medical aid stations and mobile teams to ensure rapid access to victims at any location within the event. Recent experience supports the deployment of strategically distributed advanced medical posts (AMPs), capable of performing triage, initial stabilization, ALS, and evacuation decisions.^45^

AMPs must be sized to manage both critical emergencies and low-complexity complaints, thereby preventing overload of ambulances and hospital systems.

#### 6.1.3 Communication and Command

A medical command center (MCC) should integrate all information regarding incidents, available resources, team locations, and evacuation routes. Communication failures are among the leading causes of care delays in mass gathering events.^44,46^

Practical drills with realistic simulations of critical scenarios – such as cardiac arrest occurring along the course – significantly improve team efficiency and reduce response times. Events such as the Tokyo 2020 and Beijing 2022 Olympic Games mandated simulation-based training for all professionals involved.^47^

#### 6.1.4 Event Operations

The operational phase of major sporting events requires time-dependent responses to medical emergencies within a dynamic environment involving multiple interacting factors. Complexity arises from geographic dispersion of athletes, large spectator crowds, and limited access to certain areas of the courses or sporting venues.

#### 6.1.5 Advanced Medical Posts

AMPs constitute the core of medical logistics. In marathons, primary AMPs should be installed near the start and finish areas, complemented by satellite posts along the course at high-risk locations (long ascents, heat-prone areas, zones with high spectator density). In stadium-based events, AMPs should be located behind main stands, near ambulance access points.^45,48^

#### 6.1.6 Mobile Teams

Mobile teams play a critical role in reducing response times, and several modalities can be included:^49^

- Medical cyclists: in marathons and triathlons, holding backpacks equipped with AEDs, pulse oximeters, airway supplies, and basic analgesics;
- Motorcycle ambulances: useful in congested urban areas;
- Boats or kayaks: for open-water or lake competitions;
- Pedestrian teams: in stadiums and arenas;
- 4×4 vehicles: for off-road events such as adventure races or motocross;
- AED-equipped drones: studies on the experimental use of drones delivering AEDs suggest potential reductions in time to defibrillation in outdoor simulated scenarios.^50^

A Japanese study analyzing emergencies in sports stadiums between 2015 and 2019 found that clinically severe and potentially fatal incidents frequently occurred in places such as swimming pools, restrooms, and spectator seating areas. These results underscore the need for comprehensive medical emergency systems, including strategic AED distribution, to serve athletes, spectators, and staff alike.^51^

#### 6.1.7 Communication and Command Center

Operations should be centralized within an MCC, integrating communication among AMPs, mobile teams, ambulances, and referral hospitals. Standardized communication protocols are recommended to ensure clarity in the information provided. Effective communication among organizers, health services, and security is essential for incident management, reducing triage and evacuation times.^46^

### 6.2. Medical Care and Triage

#### 6.2.1. Logistics During Medical Care

Cardiac arrest must be recognized and treated within 3 minutes, with immediate AED use. A video-based analysis of cardiac arrest cases during sports activities showed 100% survival among athletes defibrillated within this time frame.^53^

ALS procedures may be initiated when trained personnel are present, but must not delay or interrupt chest compressions, AED use, or the decision to transport the athlete. Intravenous (IV) or intraosseous (IO) epinephrine (1 mg) and amiodarone (300 mg) should be administered after the third shock if the athlete remains unresponsive prior to field removal. If the rhythm converts from shockable to non-shockable for more than one CPR cycle, immediate transfer to hospital should be considered. When moving the patient is difficult, minimizing CPR interruption is critical, and continuing resuscitation on site (even after 3 shocks) may be preferable.

#### 6.2.2. Documentation and Quality Indicators

All care must be documented using standardized medical records, including symptom onset time, time to first contact, interventions performed, and outcomes. These data enable calculation of indicators such as mean time to AED application and hospital transport rates, which are essential for post-event audits.

### 6.3. Medical evacuation

#### 6.3.1. Evacuation Criteria

Medical evacuation decisions at sporting events should consider clinical severity, feasibility of initial on-site stabilization, and availability of specialized hospital resources. International field triage guidelines recommend priority transport for patients with higher severity criteria, such as:^55,56^

- Altered mental status with Glasgow Coma Scale score ≤ 13;
- Hemodynamic instability or shock unresponsive to initial measures;
- Need for definitive referral hospital-based interventions (eg, catheterization for acute myocardial infarction).

#### 6.3.2. Evacuation Routes

Close coordination with post-cardiac arrest care teams is essential. Transport time to predefined destinations should be minimized. Dedicated evacuation routes must be planned in advance, avoiding intersections with athletes or spectator crowds. Interception points along the courses are recommended to facilitate rapid ambulance access. In Japan, a recent study highlighted that lack of predefined routes was a major cause of evacuation delays in mass sporting events.^51^

#### 6.3.3. Integration with Referral Hospitals

Referral hospitals should be notified in advance about the event, with predefined designation of units for cardiovascular, trauma, and pediatric cases. Structured prehospital notification, including updates on patient progress, allows receiving teams (who should be familiar and experienced with standardized protocols) to prepare appropriately and reduce time to intervention.^46^

#### 6.3.4 Safe Transport

Transport should occur only after initial stabilization of vital functions, except in situations where definitive treatment is hospital-based (eg, catheterization for ST-elevation myocardial infarction). Current guidelines discourage transport during ongoing CPR due to reduced effectiveness and increased risk of injury to rescuers performing maneuvers inside a moving vehicle; ideally, transport should occur after the victim has regained an organized rhythm. Transport during active resuscitation should be considered only when mechanical compression devices are available or when distances are minimal. Manual CPR in a moving ambulance compromises quality and exposes staff to unnecessary risk. In cases of recurrent cardiac arrest during transport, resuscitation may continue using air or ground ambulances with trained personnel, preferably using automated chest compression devices.

In suspected severe hyperthermia (> 40 °C), immediate cooling (cold-water immersion or ice towels) should precede transport ("cool first, transport second").

#### 6.3.5. Monitoring During Transport

- Continuous ECG monitoring in cardiovascular patients;
- Pulse oximetry, temperature, and serial blood pressure measurements for all transports;
- Capnography in ventilated patients;
- Continuous communication with the receiving hospital.

Following cardiac arrest, mild hypothermia may occur in comatose patients; active rewarming should be avoided. Hypothermia should be maintained, with targeted temperature management (32 to 36 °C) for 12 to 24 hours and active fever prevention (≤ 37.5 °C) for 72 hours. Cooling devices with temperature feedback control are preferred over prehospital cold IV fluid infusion due to the risk of recurrent arrest and pulmonary edema.

### 6.4. Legal and Safety Aspects

It is essential to document consent or refusal of transport, ensure confidentiality of clinical data in accordance with the Brazilian General Data Protection Regulation, and guarantee operational safety of evacuation routes by preventing interference from crowds or urban traffic.^48^

Knowledge transfer among organizers, medical teams, emergency services, and referral hospitals is a key element in continuous improvement of care logistics.

A recent study at the Athens Marathon showed that the absence of clear protocols shared between institutions increased the risk of duplicated efforts, communication failures, and delays during critical evacuations. Implementation of prior interinstitutional meetings, joint simulations, and daily briefings during the event was associated with improved operational efficiency and reduced response times.^46^

Another illustrative example is the field hospital model used at the 2023 Ryder Cup, where sports physicians, cardiologists, orthopedists, and emergency physicians worked side by side in a modular care system. This structure enabled real-time data collection, later used to assess areas for improvement, such as refining ambulance positioning, medication stock management, and hyperthermia training.^45^

Post-event audits, including clinical outcome reports and process indicators (time to defibrillation, on-site initiation of cooling for exertional heat stroke, proportion of cases stabilized on site vs. transferred), are indispensable tools for providing feedback to the planning of future competitions.^44^

### 6.5. Considerations by environment

- Clubs: prioritize AED placement in sports areas, with access outside training hours;
- Large arenas: separate plans for athletes and spectators; resources coordinated by a medical director;
- Marathons and mass events: up to 5 first responders per 1,000 athletes; at least 1 ALS-trained physician per 2,500 runners; aid stations approximately every 1.5 km; ambulances **preferentially positioned in the second half of the course**;
- Olympic Games: multi-venue logistics; need for mobile teams and medical interpreters; joint training to standardize responses (Figure 5).

**Figure 5:** Specifics by environment. AED: automated external defibrillator; ALS: advanced life support; BLS: basic life support; SCA: sudden cardiac arrest.

## 7. Basic and Advanced Life Support Protocols (the Chain of Survival) Adapted to Sports Settings

Cardiac arrest in athletes, although uncommon, is a catastrophic event that requires an immediate, coordinated, and efficient response. Adapting the AHA "Chain of Survival" to the sports environment is essential to improve outcomes.^25,56-59^

### 7.1. Immediate Recognition and Activation of Emergency Medical Services

- Sports-specific adaptation: sudden collapse may be difficult to distinguish from other conditions (eg, extreme fatigue, seizure, trauma). **The guiding principle is to treat any collapsed, unresponsive athlete as having cardiac arrest until proven otherwise**;
- Action: Check responsiveness (shake shoulders and shout). If there is no response, call for help loudly and immediately activate the local EMS (SAMU 192 in Brazil). Assign a specific individual to place the call. Request an AED. In sports venues, the AED must be accessible and signposted, with an ideal access time of less than 3 minutes.

### 7.2. Early, High-Quality Cardiopulmonary Resuscitation

- Sports-specific adaptation: CPR may be initiated immediately by trained individuals present on site (coaches, athletic trainers, trained teammates, referees, or even athletes). The technique follows standard AHA and SBC recommendations;
- Action (basic):
  1. Open the airway (head-tilt/chin-lift maneuver);
  2. Check breathing; if absent or agonal gasping is present, begin chest compressions;
  3. Chest compressions at a rate of 100–120/min, with a depth of 5–6 cm, allowing complete chest wall recoil. Minimize interruptions;
  4. Ventilations: if trained and willing, provide a 30:2 compression-to-ventilation ratio; if untrained or unwilling, perform continuous chest compressions only.

### 7.3. Rapid Defibrillation

- Sports-specific adaptation: VF is the most common cause of sudden cardiac arrest in athletes. Shock administration is the only effective treatment. AED should be applied within 3 minutes;
- Action (basic/advanced):
  1. Turn on the AED and follow voice prompts.
  2. Expose the chest and quickly dry excessive sweat, if necessary, to ensure proper electrode adhesion.
  3. Ensure that no one is touching the victim during rhythm analysis and shock delivery; administer the shock if advised and immediately resume CPR.

### 7.4. Advanced Life Support and Post-Cardiac Arrest Care

- Sports-specific adaptation: provided by the on-site EMS team, while the host sports organization must facilitate ambulance access and ensure transfer to a predesignated referral hospital;
- Action (advanced – by health care professionals):
  1. Advanced airway management: endotracheal intubation or supraglottic airway placement to ensure adequate oxygenation and ventilation;
  2. Vascular access: establish IO or IV access for medication administration;
  3. Pharmacologic therapy: administer epinephrine (1 mg) IV/IO every 3–5 minutes during CPR. If VF/VT persists, consider amiodarone (300 mg) IV/IO;
  4. Management of reversible causes – the 5Hs (hypovolemia, hypoxia, hypothermia, hydrogen ion/acidosis, hypo-/hyperkalemia) and 5Ts (toxins, tamponade [cardiac], tension pneumothorax, thrombosis [coronary], thromboembolism [pulmonary]): in sports settings, one should strongly suspect:
    – *Commotio cordis*: blunt chest trauma (eg, ball impact). The treatment is immediate defibrillation;
    – Hypothermia/hyperthermia: in endurance sports;
    – Electrolyte disturbances: severe dehydration.
  5. Post-resuscitation care: after ROSC, initiate ventilatory support, targeted temperature management (32–36 °C – therapeutic hypothermia), and rapid transport to a hospital with percutaneous coronary intervention capability and comprehensive care.

### 7.5. Site-specific protocol for training and competition venues

1. Prevention: cardiovascular screening (medical history and physical examination) to identify athletes at increased risk;
2. Preparation: ensure the presence of a trained response team and the availability of a functional AED with charged batteries. A written action plan must be in place and known to all staff;
3. Simulation: conduct regular training sessions and simulation drills (every 6 months) involving all personnel, from security staff to physicians.

## Data Availability

The data underlying the text of the Position Statement are included in the manuscript.

## List of abbreviations and acronyms

ACLS: Advanced cardiac life support
AED: Automated external defibrillator
AHA: American Heart Association
ALS: Advanced life support
AMP: Advanced medical post
AVC: Arrhythmogenic ventricular cardiomyopathy
BLS: Basic life support
CAD: Coronary artery disease
CPR: Cardiopulmonary resuscitation
EACPR: European Association of Cardiovascular Prevention and Rehabilitation
EAP: Emergency action plan
ECG: Electrocardiogram
EMS: Emergency Medical Services
ERC: European Resuscitation Council
ESC: European Society of Cardiology
HCM: Hypertrophic cardiomyopathy
HEPA: High-efficiency particulate air
ICU: Intensive care unit
IO: Intraosseous
IV: Intravenous
MAP: Medical action plan
MCC: Medical command left
PPE: Preparticipation physical evaluation
ROSC: Return of spontaneous circulation
SADS: Sudden arrhythmic death syndrome
SBC: Brazilian Society of Cardiology (*Sociedade Brasileira de Cardiologia*)
SBME: Brazilian Society of Sports Medicine (*Sociedade Brasileira de Medicina do Esporte*)
SCA: Sudden cardiac arrest
SCD: Sudden cardiac death
TECA: Brazilian advanced cardiovascular emergency training (*treinamento de emergências cardiovasculares avançado*)
VF: Ventricular fibrillation
VT: Ventricular tachycardia
